# Identification of cuproptosis-related long noncoding RNA signature for predicting prognosis and immunotherapy response in bladder cancer

**DOI:** 10.1038/s41598-022-25998-2

**Published:** 2022-12-10

**Authors:** Gaomin Huang, Yawei Huang, Chiyu Zhang, Yi Jiang, Zhenfeng Ye, Chen He, Fanfan Yu, Zitong Chen, Xiaoqing Xi

**Affiliations:** 1 Department of Urology, Second Affiliated Hospital of Nanchang University, Nanchang, 330006 China; 2 Jiangxi Key Laboratory of Molecular Medicine, Second Affiliated Hospital of Nanchang University, Nanchang, 330006 China; 3 Department of Obstetrics and Gynecology, First Affiliated Hospital of Nanchang University, Nanchang, 330006 China

**Keywords:** Bladder cancer, Cancer microenvironment

## Abstract

Bladder cancer (BC) is the most common malignant tumour of the urinary system and one of the leading causes of cancer-related death. Cuproptosis is a novel form of programmed cell death, and its mechanism in tumours remains unclear. This study aimed to establish the prognostic signatures of cuproptosis-related lncRNAs and determine their clinical prognostic value. RNA sequencing data from The Cancer Genome Atlas were used to detect the expression levels of cuproptosis-related genes in BC. Cuproptosis-related lncRNAs linked to survival were identified using co-expression and univariate Cox regression. Furthermore, consensus cluster analysis divided the lncRNAs into two subtypes. Subsequently, we established a signature model consisting of seven cuproptosis-related lncRNAs (AC073534.2, AC021321.1, HYI-AS1, PPP1R26-AS1, AC010328.1, AC012568.1 and MIR4435-2Hg) using least absolute shrinkage and selection operator regression. Survival analysis based on risk score showed that the overall survival and progression-free survival of patients in the high-risk group were worse than those in the low-risk group. Multivariate Cox analysis demonstrated the independent prognostic potential of this signature model for patients with BC. Moreover, age and clinical stage were also significantly correlated with prognosis. The constructed nomogram plots revealed good predictive power for the prognosis of patients with BC and were validated using calibration plots. Additionally, enrichment analysis, Single sample gene set enrichment analysis and immune infiltration abundance analysis revealed significant differences in immune infiltration between the two risk groups, with high levels of immune cell subset infiltrations observed in the high-risk group accompanied by various immune pathway activation. Moreover, almost all the immune checkpoint genes showed high expression levels in the high-risk group. Moreover, TIDE analysis suggested that the high-risk group was more responsive to immunotherapy. Finally, eight drugs with low IC50 values were screened, which may prove to be beneficial for patients in the high-risk group.

## Introduction

Bladder cancer (BC) is one of the most common malignant tumours of the urinary system, with high morbidity and mortality rates worldwide. The proportion of male patients with BC is higher than that of females, wherein men with BC have the fourth highest incidence and eighth highest mortality rate among all cancers^1^. BC can be divided into two types: non-muscular invasion BC (NMIBC) and muscular invasion BC (MIBC), based on the presence ofmuscular invasion^2^. With the emergence of targeted therapy and immunotherapy, patients with BC requiringneoadjuvant therapy have more combinatorial options based on traditional surgical approaches^3,4^. The combination of multiple treatment strategies prolongs patient survival to some extent; however, the prognosis remains poor and the overall recurrence and mortality rates for patients with BC remain high^5^. Therefore, the exploration of potentially effective prognostic biomarkers and molecular signatures is crucial for early diagnosis and clinical decision-making, and to aid in the further development of new therapies.

Recently, cuproptosis has been reported as a novel copper-dependent form of programmed cell death^6^. Copper (Cu) causes lipid-acylated protein aggregation and subsequent iron–sulfur cluster protein loss by directly binding to the lipid-acylated component of the tricarboxylic acid (TCA) cycle. This leads to proteotoxic stress and ultimately cell death^7^. As an essential mineral, Cu participates in various biological processes, including energy metabolism and signal transduction^8^. Current studies report that Cu levels are significantly altered in various malignant tumours, with higher levels in most tumours than normal tissues^9–12^. The dysregulation of Cu homeostasis plays an important role in the development, progression and metastasis of cancer. Cu acts as a tumour promoter and stimulates cancer cell proliferation and tumour growth^13^. A significant correlation between clinical stage and Cu levels in colorectal cancer has been reported^14^. Additionally, Cu exposure is involved in the occurrence of prostate cancer and could be a potential risk factor for carcinogenesis^15^. Furthermore, Cu plays a specific role in cancer progression and resistance to oncology drugs, and Cu chelators are reported to have anti-tumour and anti-metastatic benefits^16^. Currently, some metal-binding compounds, including Cu, have shown broad prospects in targeted therapy for tumours^17,18^. Thus, targeting Cu could be a novel approach in cancer therapy^19^.

Long non-coding RNAs (lncRNAs) are a class of non-protein coding RNAs (ncRNAs) having a length of more than 200 nucleotides, which perform various biological functions in eukaryotes, involving mRNA stability, transcriptional regulation and translation^20^. Numerous studies have reported that lncRNAs are closely related to tumorigenesis, metastasis and tumour immunity^21–23^. Additionally, it is a key regulatory factor in the development and progression of BC and can be used as a potential biomarker and therapeutic target^24^. Recently, it has been reported that the overexpression of lncRNA SOX2OT is associated with poor prognosis in BC. SOX2OT knockdown inhibited tumour growth and reduced metastasis in vivo^25^. Furthermore, lncRNA CASC9 has been reported as a carcinogenic lncRNA that activates the Wnt/β-catenin signalling pathway via the ceRNA mechanism, thereby playing an oncogenic role in BC^26^. Another study showed that lncRNA-LET reversed gemcitabine chemoresistance in BC, with reduced lncRNA-LET levels predicting poor prognosis in patients with BC^27^. Additionally, lncRNAs also regulate the PD-1/PD-L1 pathway of tumour cells and participate in immune response and immunotherapy^28^. The prognostic profile of lncRNAs has been widely reported in BC owing to their key role in tumours^29,30^. However, the role of cuproptosis-related lncRNAs in the prognosis and tumour immune microenvironment of BC remains unexplored.

Therefore, this study explores the role of cuproptosis-related lncRNAs in BC, which can contribute to the calcification of the potential association between cuproptosis and lncRNAs with immunotherapy. Public databases were used to mine lncRNAs overexpressed by cuproptosis genes to study the prognosis and immunological significance of cuproptosis-related lncRNAs. Subsequently, a prognostic feature of cuproptosis-related lncRNAs was established to predict survival in patients with BC, which provides novel ideas for exploring new molecular prognostic markers and potential therapeutic targets for BC.

## Materials and methods

### Data collection

RNA-seq data and relevant clinical information were obtained from The Cancer Genome Atlas database (TCGA, GDC (cancer.gov)) on 17 April 2022, which contained 19 normal control samples and 412 BC samples. The FPKM value matrix for RNA-SEQ data provided on the official website was used for subsequent analysis. Mutation data of patients with BC were downloaded from TCGA database in MAF format. A total of 19 cuproptosis genes were extracted from published literature^7^ and are detailed in Table S1.

### Screening for co-expressed lncRNAs of cuproptosis-related genes

Limma software package in R software (version 4.1.1, r-project.org) was used to extract the expression levels of 19 cuproptosis genes from TCGA dataset^31^. The expressions of tumour tissue and normal tissues were visualised using the pheatmap package to label genes with *P* values < 0.05. Firstly, the limma package was used to screen for lncRNAs that were significantly associated with cuproptosis-related genes, with the filtering criteria of correlation coefficient set at |Pearson R|> 0.4 and *P* < 0.001. Following this, dplyr, GGalluvial and GGplot2 packages were used to plot Sankey maps of cuproptosis and its associated lncRNAs. Additionally, univariate Cox regression analysis was performed using the survival package to select prognosis-related lncRNAs (*P* < 0.01) and forest maps were generated. Finally, the differential expression of lncRNAs related to prognosis was visualized using pheatmap. Furthermore, Reshape2 and GGPUBR packages were used to draw heatmaps and boxplots.

### Consensus cluster analysis and immunological correlation

To explore the molecular signatures of cuproptosis-related lncRNAs with prognostic value in BC, ConsensusClusterPlus and limma package were used^32^. BC samples were classified into two different subtypes and survival differences among different subtypes were analysed. Additionally, Pheatmap package was used to plot a heatmap of the different clusters in correlation with clinicopathological features. Furthermore, the expression of immune checkpoint genes was compared across subtypes along with the correlation of immune checkpoint molecules with cuproptosis-related lncRNAs.

### Establishment and validation of signature model

We randomly divided the TCGA cohort into the training group and the test group in a 1:1 ratio and assessed the degree of difference in clinicopathological features between the two groups. The least absolute shrinkage and selection operator (LASSO) algorithm was used to perform regression analysis on the training group. The penalty parameter (λ) of the least criteria ten-fold cross-validation was used to select the model, and seven genes and their coefficients were determined to establish cuproptosis-related lncRNA signature models. Based on the normalized expression of each lncRNA (Expr) and its regression coefficient (Coef) risk score was calculated as follows: Risk Score = ${\sum }_{i}^{n}\mathrm{Expri }\times \mathrm{Coefi}$. According to the median risk score, TCGA cohort was classified into low-risk and high-risk groups. Kaplan–Meier curves were plotted using the survival package to compare survival differences between the low- and high-risk groups, and heatmaps were used to demonstrate expression differences of cuproptosis-related lncRNAs between the low- and high-risk groups. Moreover, the accuracy of the prognostic model was validated using a test cohort and an independent GEO dataset (GSE31684)^33^.

### Prognostic value of risk signature

Univariate and multivariate Cox regression were used to analyse the prognostic value of risk scores and clinicopathological parameters and independent risk prognostic factors. Time-dependent receiver operating signature (ROC) curves and area under curve (AUC) values for 1-, 3- and 5-year survival were used to assess the predictive power of prognostic signatures. A nomogram plot was constructed based on the multivariate Cox regression analysis. Moreover, the consistency index and calibration curve were used to evaluate the consistency of the risk profile model using the sample information. The relationship between each clinicopathological parameter and risk score was evaluated based on the clinical information of patients in TCGA cohort, including age, sex, tissue grade and clinical stage. Furthermore, survival differences among different risk groups stratified by clinicopathological parameters were explored in our regression model.

### Enrichment analysis of risk prognostic signature

Patients in TCGA cohort were divided into two subgroups based on the median risk score. The ‘limma’ R package was run to screen for differential genes between the low-risk and high-risk groups according to the following criteria: |log 2 FC|≥ 1.5 and FDR < 0.05. The ‘clusterProfiler’ package was then applied for Gene Ontology (GO) and Kyoto Encyclopedia of Genes and Genomes (KEGG) enrichment analysis and visualization^34,35^.

### Tumour immune microenvironment of risk prognostic signature

Based on the enrichment results, we evaluated immune cell infiltration in TCGA dataset using seven algorithms, namely XCELL, TIMER, QUANTISEQ, MCPCOUNTER, EPIC, Cibersort-ABS and CIBERSORT. Spearman correlation analysis was used to explore the correlation between immune cell subsets and risk scores. The ESTIMATE algorithm was then used to calculate the immunity scores, including immunity, stromal and ESTIMATE scores for the different risk groups. The ‘GSVA’ package was used to perform ssGSEA, which calculated the score of infiltrating immune cells and assessed the activation of immune-related pathways^36^. The immune cell and pathway scores in the low- and high-risk groups were presented via a boxplot. Additionally, we compared the expression of immune checkpoint-related genes in different risk groups.

### Immunotherapy for prognosis signatures

TIDE analysis was used to predict the potential efficacy of immunotherapy in different risk subgroups. The ‘pRRophetic’ package was then used to predict the sensitivity of patients with BC in the high- and low-risk groups to various oncology drugs, which was expressed as a semi-maximum inhibitory concentration (IC50)^37^.

### Real-time PCR

We collected bladder cancer and adjacent tissues from the Second Affiliated Hospital of Nanchang University from September 2021 to February 2022. RT-PCR was performed using Green qPCR Super Mix (Transgen, AQ601, Beijing) with fluorescent quantitative Real-Time PCR System (Applied Biosystems, 7900H, USA) according to the instructions. We normalized the relative lncRNA expression levels to B-actin, respectively. The sequences of primers (5′–3′) in this study were listed as follows: MIR4435-2HG, forward primer: GCCAGGACACAGCCATCTAAAGC, reverse primer: TTCCTCAGCATGGTGTGGTTCATTC; PPP1R26-AS1, forward primer: CGTGAAGGGCTTTAGGAAGGAGAAC, reverse primer: TCCACCACCACCAGACCAAGG; B-actin, forward primer: CACCATTGGCAATGAGCGGTTC, reverse primer: AGGTCTTTGCGGATGTCCACGT.

### Ethics statements

All methods were carried out in accordance with relevant guidelines and regulations. All experimental protocols were approved by the Ethics Committee of the Second Affiliate Hospital of Nanchang University (No. Review [2021] No. (115)), China. All of the human tissues used in the present study were obtained with written informed consent from all subjects and their legal guardians.

### Statistical analysis

We utilized the Wilcox rank-sum test for comparing mRNA and lncRNA expression levels between the two groups, and the Chi-square test was used to compare categorical variables. Kaplan–Meier and log-rank analyses were used to compare overall survival (OS) between different groups. Univariate and multivariate Cox regression was used to determine the independent prognostic value of risk models. The correlations among subtypes, clinicopathological features, risk score, immune checkpoint expression and immune infiltration level were examined using Pearson’s correlation test. All statistical analyses were performed using R software (version 4.1.1). *P* < 0.05 were considered statistically significant.

## Results

### Screening of cuproptosis-related LncRNAs

**Figure 1:**
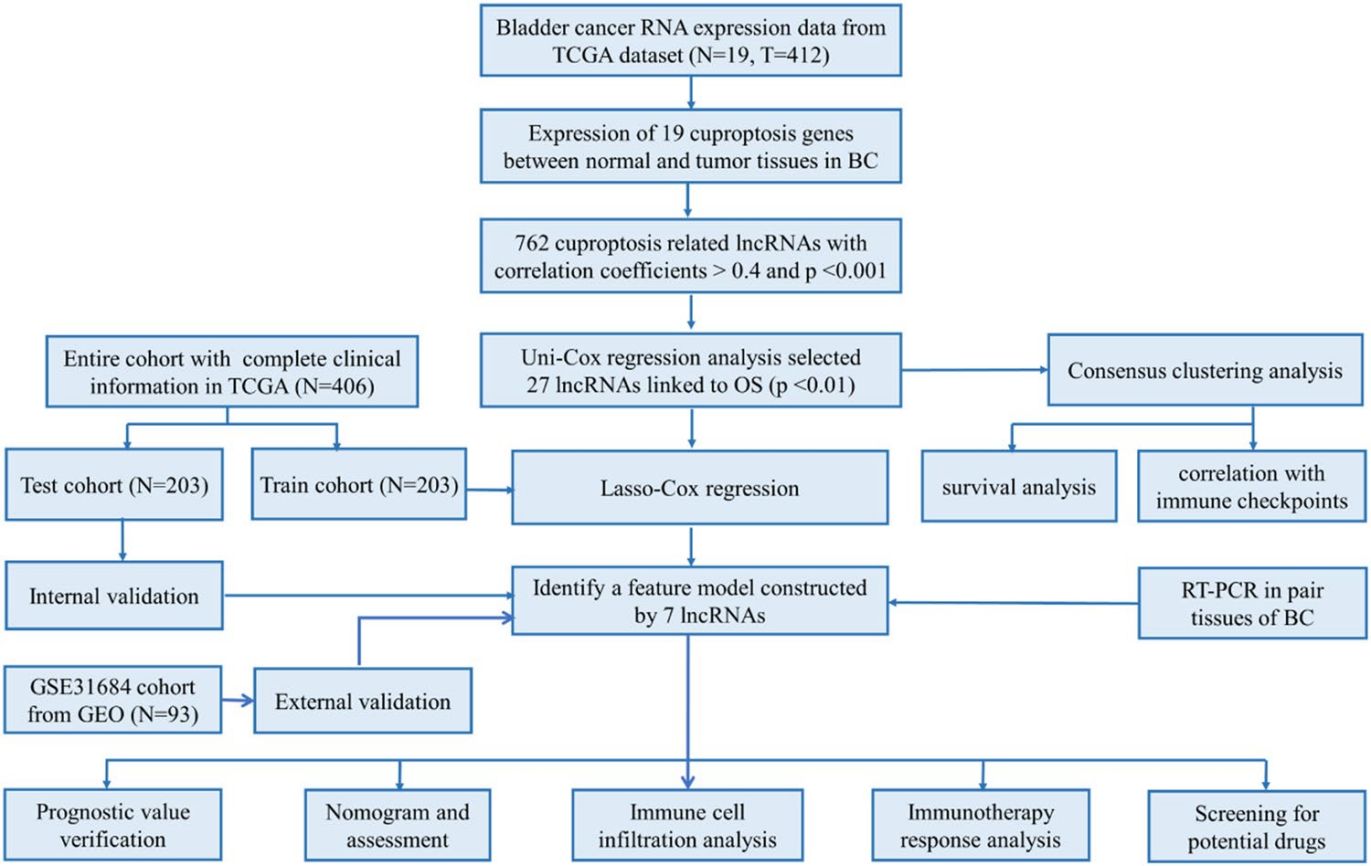
Overall flow chart of the study.

**Figure 2:**
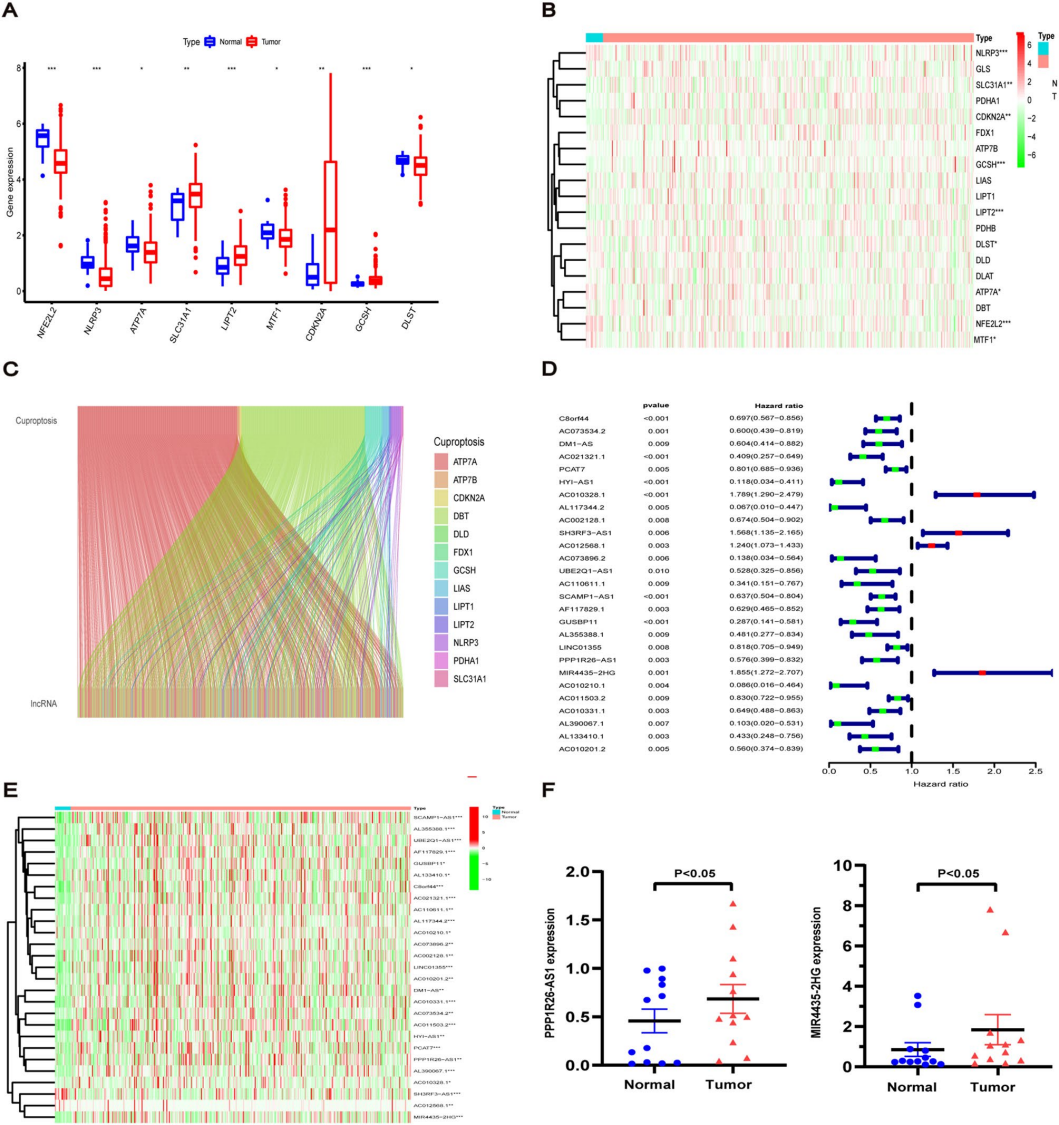
Identification of cuproptosis-related lncRNAs in BC. (**A**) Differential expression of 19 cuprotosis genes between BC tissues and normal tissues. (**B**) The heatmap of 19 cuprotosis genes with color green for low-expression and color red for high-expression. (**C**) Sankey relational plot for cuprotosis genes and cuproptosis-related lncRNAs. (**D**) Forest plot of 27 cuproptosis-related lncRNAs identified to be significantly correlated with prognosis. (**E**) The heatmap of 27 cuproptosis-related lncRNAs associated with survival. (**F**) Expression of MIR4435-2Hg and PPP1R26-AS1 in 12 pair of bladder cancer and adjacent tissues. *P < 0.05, **P < 0.01, and ***P < 0.001.

The flow chart of the study design is presented in Fig. 1. Transcriptomic data from 412 BC tissues and 19 normal control tissues revealed that four genes were upregulated and five genes were downregulated in tumour tissues (Fig. 2A). Then, heatmaps aided in the visualisation of their expression (Fig. 2B). Based on the expression of 19 cuproptosis genes, the expression levels of cuproptosis-related lncRNAs were extracted. The filtering conditions were set as correlation coefficients > 0.4 and *P* < 0.001. Finally, 762 co-expressed lncRNAs were obtained (Fig. 2C), which are detailed in Table S2. Then, univariate cox regression analysis was performed on these lncRNAs, and 27 cuproptosis-related lncRNAs were identified to be significantly correlated with prognosis, which was visualized using heatmap (Fig. 2D,E). Finally, we verified the differential expression of MIR4435-2Hg and PPP1R26-AS1 in bladder cancer and adjacent paired tissues. The results showed that MIR4435-2Hg and PPP1R26-AS1 were highly expressed in bladder cancer tissues (Fig. 2F). The experimental results were consistent with the TCGA dataset.

### Consensus clustering analysis

**Figure 3:**
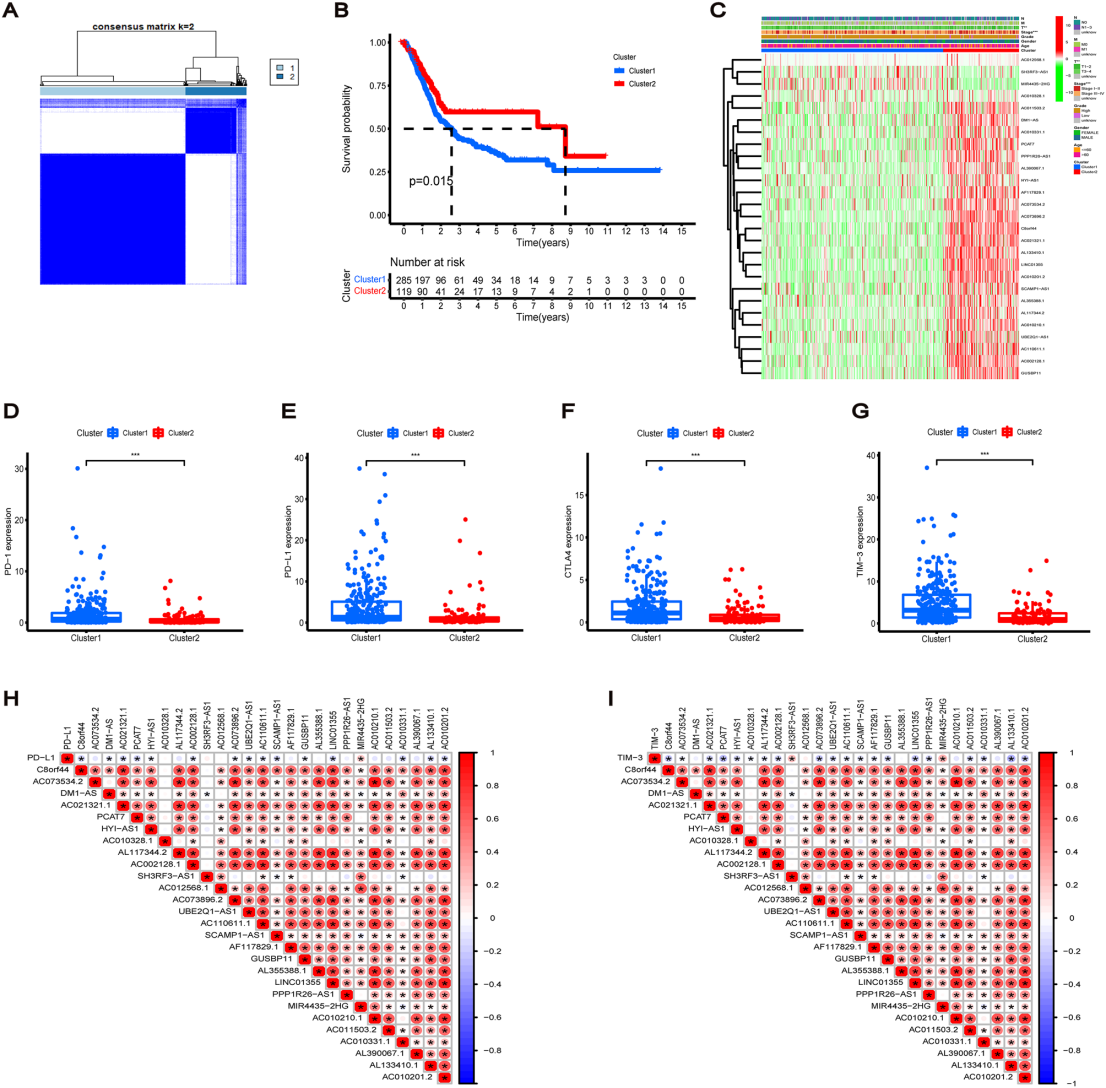
Consensus clustering analysis of 27 cuproptosis-related lncRNAs. (**A**) BC patients were divided into 2 groups by consensus clustering. (**B**) Kaplan–Meier curves of overall survival (OS) between the two clusters. (**C**) Heatmap and the clinicopathological features of two clusters, including gender, age, T, M, N status and pathological grade. (**D**–**G**) Differential expression of immune checkpoint genes (PD-1, PD-L1, CTLA-4 and TIM-3) in the two clusters. (**H**,**I**) The correlation of immune checkpoint genes (PD-L1 and TIM-3) with 27 cuproptosis-related lncRNAs linked to survival. *P < 0.05, **P < 0.01, and ***P < 0.001.

To explore the association between lncRNAs and different subtypes of BC, we grouped 412 patients with BC using consensus clustering. Increasing the clustering variable (k) from 2 to 10, the samples were divided into two well-distinguishable groups (Fig. 3A). The OS of the two clusters was significantly different, with Cluster 1 having a worse prognosis (*P* = 0.015, Fig. 3B) than Cluster 2. The heatmap presented the differential expression of cuproptosis-related lncRNAs between Cluster 1 and Cluster 2, and the clinicopathological signatures of the two subgroups such as gender, age, pathological TMN status and tumour grade were compared. The clinical stage and pathological T status of the two Clusters differed in the heatmap, and the grouping of consistent clusters was closely related to clinicopathological features (Fig. 3C). Next, we detected the differential expressions of immune checkpoint genes (PD-1, PD-L1, CTLA-4 and TIM-3) in the two clusters. The expression levels of PD-1, PD-L1, CTLA-4 and TIM-3 were higher in Cluster 1 than that in Cluster 2 (Fig. 3D–G). The expression of cuproptosis-related lncRNA was significantly correlated with immune checkpoint (Fig. 3H,I). These results suggest that cuproptosis-related lncRNAs could be involved in the immunotherapy of BC.

### Construction of risk signature model in TCGA cohort

**Figure 4:**
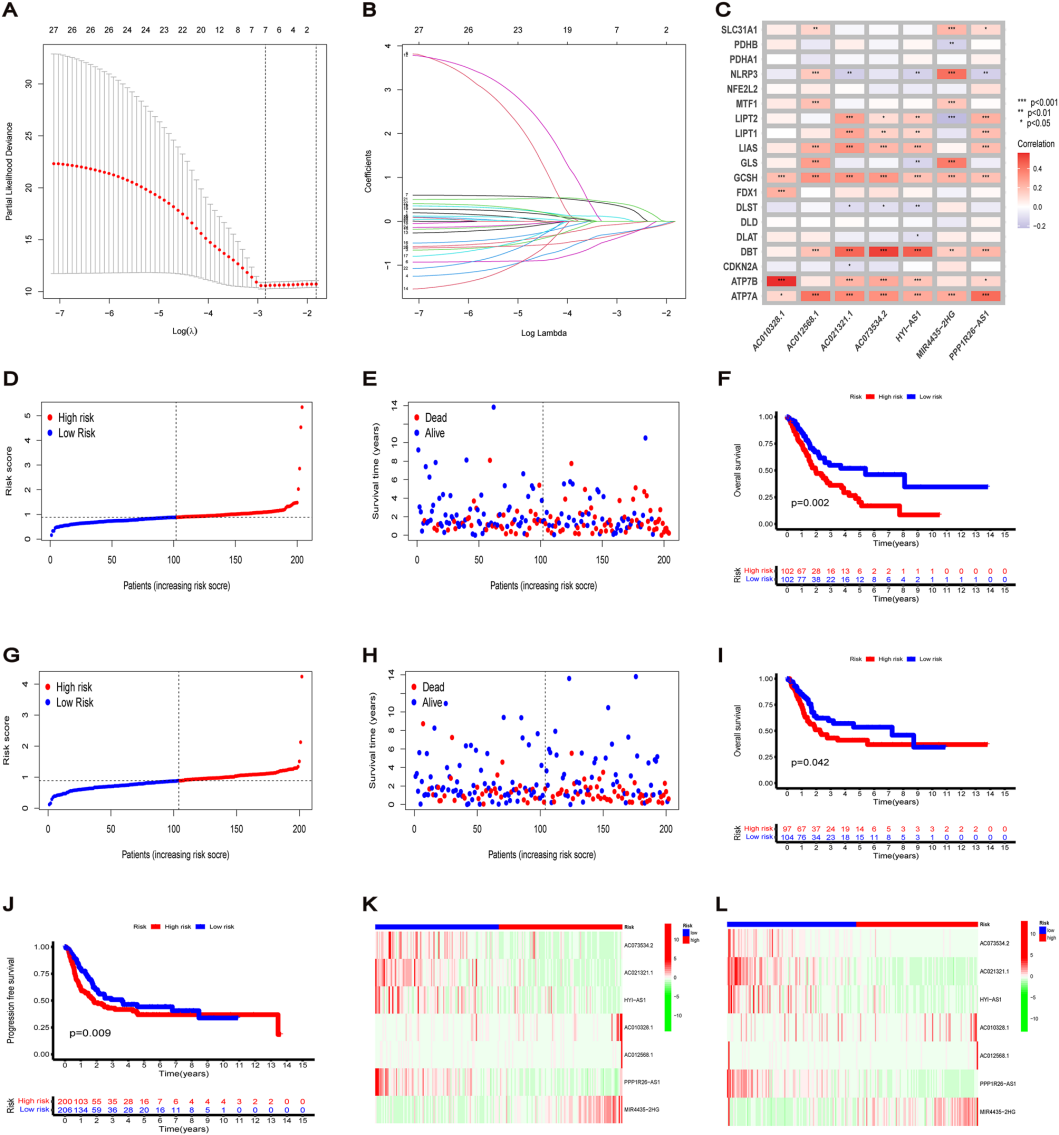
Construction of prognostic signature for cuproptosis-related lncRNAs. (**A**) The tenfold cross-validation for variable selection in LASSO regression for the training group cohort. (**B**) 7 lncRNAs and their coefficients were determined by LASSO algorithm. (**C**) The heatmap of the correlation between cuproptosis gene and lncRNAs. (**D**,**G**) BC patients in train and test cohort divided into low-risk and high-risk subgroups according to the median risk score. (**E**,**H**) The survival status of each patient with different risk in train and test cohort. (**F**,**I**) Kaplan–Meier curves of OS in different risk groups of train and test cohort. (**J**) Progression free survival for the overall sets between high-risk group and low-risk group (**K**,**L**) The heatmap of 7 cuproptosis-related lncRNAs with different risk subgroups in train and test cohort.

A cohort of 406 patients with BC having complete survival-related and clinical information in TCGA was randomly divided into a training and a test group in a 1:1 ratio, and no significant bias was observed for each parameter of the clinicopathological signatures in both groups. A risk signature model consisting of seven lncRNAs was constructed based on LASSO regression analysis for the training group (Fig. 4A,B). The risk score was calculated as follows: Risk Score = (− 0.057* AC073534.2 exp.) + (− 0.244* AC021321.1 exp.) + (− 0.325* HYI-AS1 exp.) + (0.320* AC010328.1 exp.) + (0.060* AC012568.1 exp.) + (− 0.268* PPP1R26-AS1 exp.) + (0.391* MIR4435-2HG exp.). The heatmap of the correlation between cuproptosis-related genes and lncRNAs is shown in Fig. 4C. Furthermore, TCGA cohort was divided into low- and high-risk groups based on the median risk score (Fig. 4D,G). The survival status of patients differed by risk, with patients in the high-risk group having a higher mortality rate and shorter survival time than those in the low-risk group (Fig. 4E,H). Additionally, the Kaplan–Meier curves of both groups showed that the OS of the high-risk group was worse compared to the low-risk group. Progression-free survival for the overall cohort also suggested a worse prognosis for the high-risk group than the low-risk group (Fig. 4F,I,J). We also used GEO dataset (GSE31684) to validate the prognostic ability of the risk model (Fig. 6A–C). The heatmap showed that the expression of cuproptosis-related lncRNAs was different between the low- and high-risk groups. The expression of four lncRNAs (AC073534.2, AC021321.1, HYI-AS1 and PPP1R26-AS1) in the low-risk group was higher than that in the high-risk group, while the other three lncRNAs (AC010328.1, AC012568.1 and MIR4435-2HG) had higher expression levels in the high-risk group (Fig. 4K–L).

### Correlation of risk signature with clinicopathological parameters

**Figure 5:**
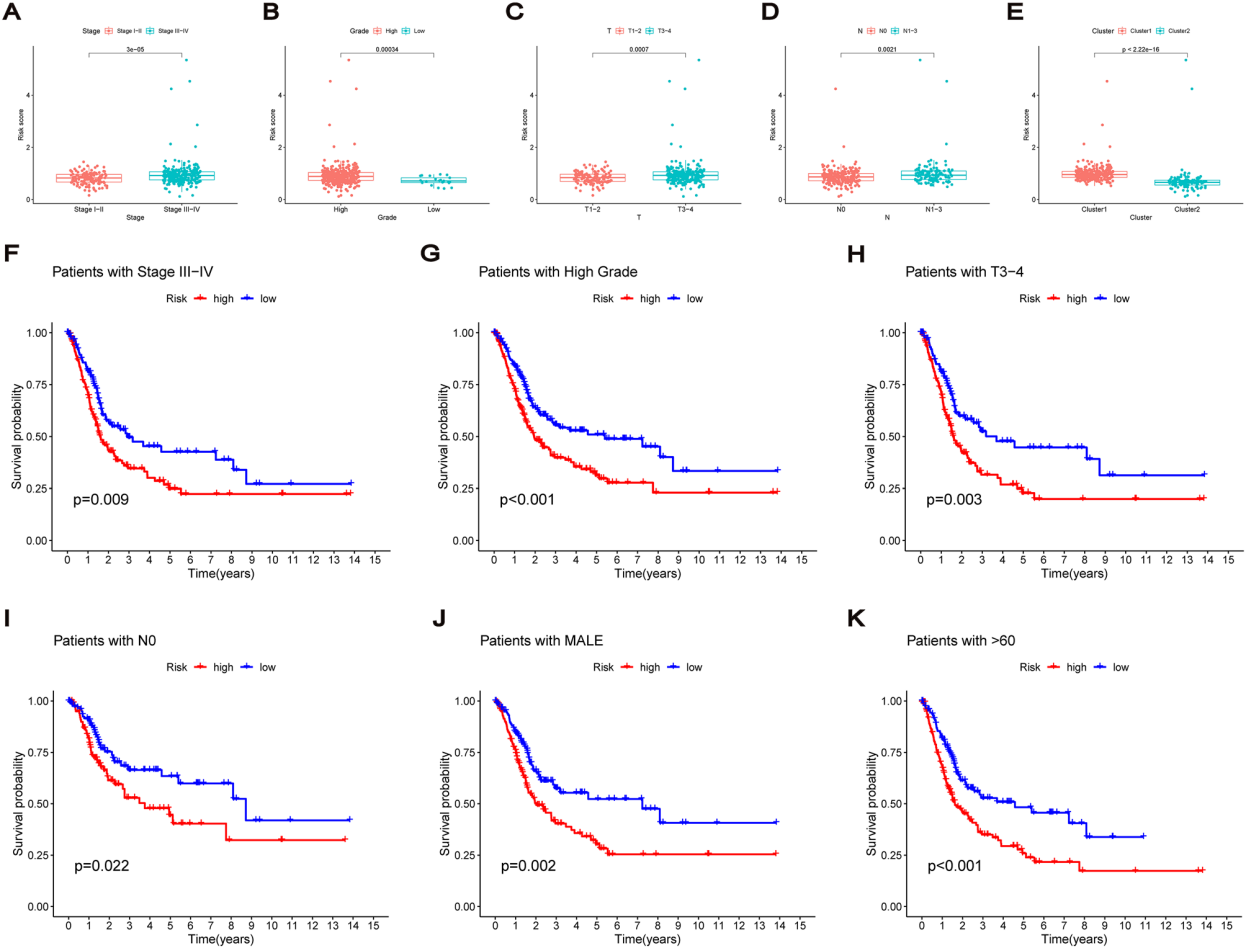
Correlation of risk signature with clinicopathological parameters. Risk score of patients with different clinicopathological stratification including (**A**) stage, (**B**) grade, (**C**) T and (**D**) N. (**E**) Risk score of patients between cluster 1 and 2. Kaplan–Meier survival curves of low-risk and high-risk groups among BC patients with (**F**) stage III–IV, (**G**) high-grade, (**H**) T3–4, (**I**) N0, (**J**) male and (**K**) age > 60.

We first stratified BC samples by age, sex, clinical stage, pathological grade, T stage, N stage and M stage to evaluate the relationship between each clinicopathological parameter and risk score. The results showed that patients with BC having a high clinical stage, high grade, lymph node metastasis and T3–4 stage had higher risk scores. Moreover, patients with Cluster 1 also had higher risk scores (Fig. 5A–E). Kaplan–Meier survival curves were then compared between the low- and high-risk groups in different clinicopathological stratifications, which revealed that the high-risk group had a worse prognosis than the low-risk group among patients with the following factors: stage III–IV, high-grade, N0, T3–4, male and age > 60 (Fig. 5F–K). Thus, the suggested risk signatures have the potential to predict the prognosis of patients with different clinicopathological stratification. Notably, high-risk patients were significantly correlated with stage, T stage, N stage and pathological grade.

### Prognostic value of risk signature

**Figure 6:**
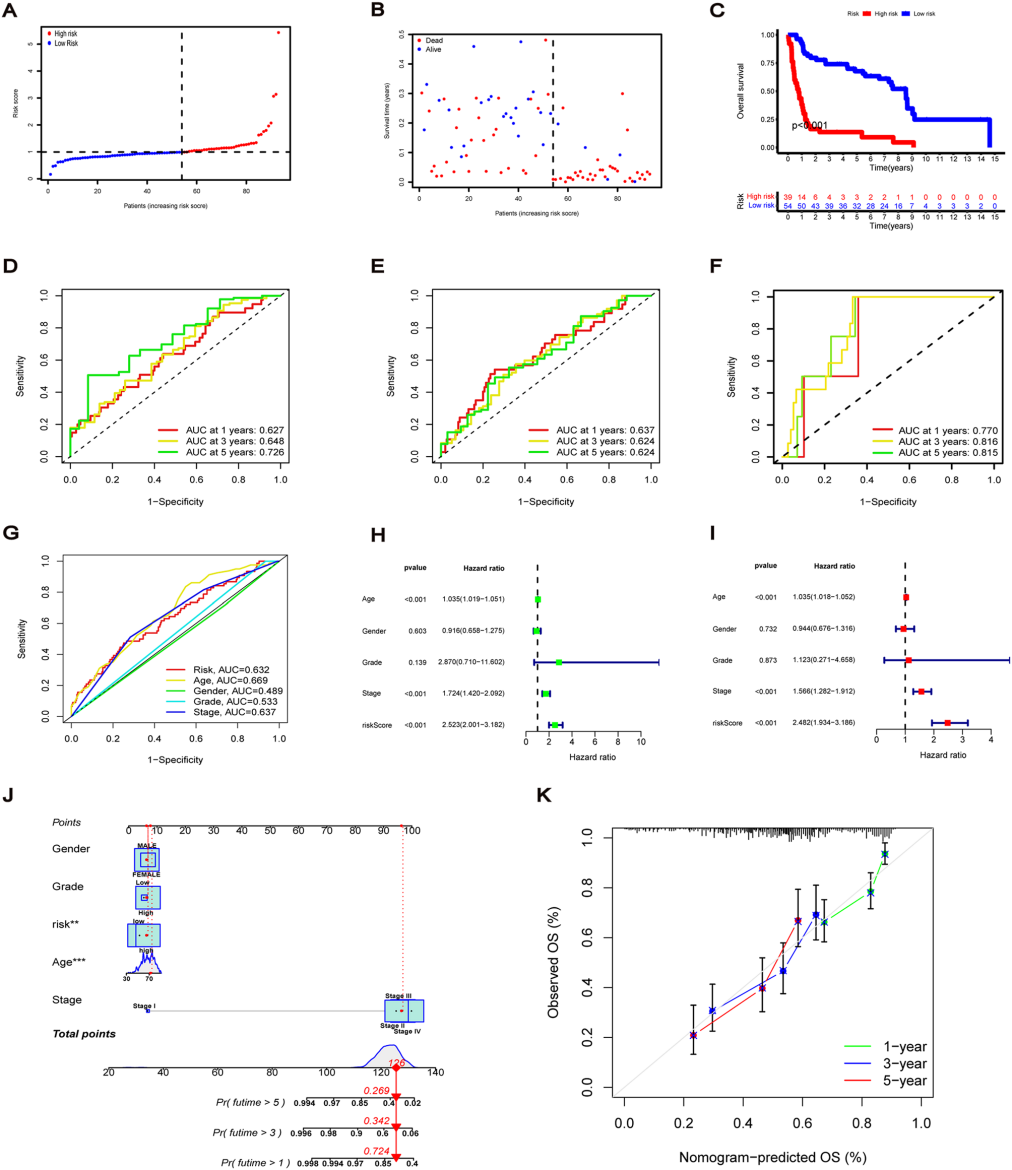
Prognostic value of risk signature. (**A**) BC patients in GSE31684 cohort divided into low-risk and high-risk subgroups according to the median risk score. (**B**) The survival status of each patient with different risk in GSE31684 cohort. (**C**) Kaplan–Meier curves of OS in different risk groups of GSE31684 cohort. (**D**–**F**) ROC curves of 1-year, 3-year, and 5-year survival rate for the risk signature in train, test and GSE31684 cohort. (**G**) ROC curves of risk score and clinicopathologic features on the 3-year. (**H**) Univariate Cox regression for risk score and the clinicopathological features. (**I**) Multivariate Cox regression for risk score and the clinicopathological features. (**J**) Nomogram for predicting 1-year, 3-year and 5-year survival rates of BC patients. (**K**) The calibration curves validate the consistency between the actual outcome and the predicted result for 1-year, 3-year and 5-year survival rates.

Time-dependent ROC was used to assess the predictive performance of risk signature models and to evaluate the AUC. The AUC of the training group showed that the 1-, 3- and 5-survival rates were 0.627, 0.648 and 0.726, respectively, while that of the test group was 0.637, 0.624 and 0.624, respectively (Fig. 6D,E). And that the AUC of GSE154261 cohort from GEO indicated that the constructed model was reliable and robust for prognostic prediction (Fig. 6F). Furthermore, the AUC of risk score, age and clinical stage on the 3-year ROC curve of the risk model were 0.632, 0.669 and 0.637, respectively, indicating their major predictive power (Fig. 6G). Forest plots based on univariate and multivariate Cox regression analysis showed that risk score was an independent predictor of poor survival. Additionally, age and clinical stage were also considered to be independent prognostic parameters associated with poor OS (Fig. 6H,I). Based on the above results, we constructed a nomogram to predict the 1-, 3- and 5-year survival rates of patients with BC. Moreover, the predicted results for one patient are reported here (Fig. 6J). The calibration curve also demonstrated that the prediction of 1-, 3- and 5-year survival rates by the nomogram is in good agreement with the actual rates observed (Fig. 6K). These findings thus indicate that the risk signature model established has a good predictive ability for the prognosis of patients with BC.

### Principal component analysis (PCA) and tumour mutation burden

**Figure 7:**
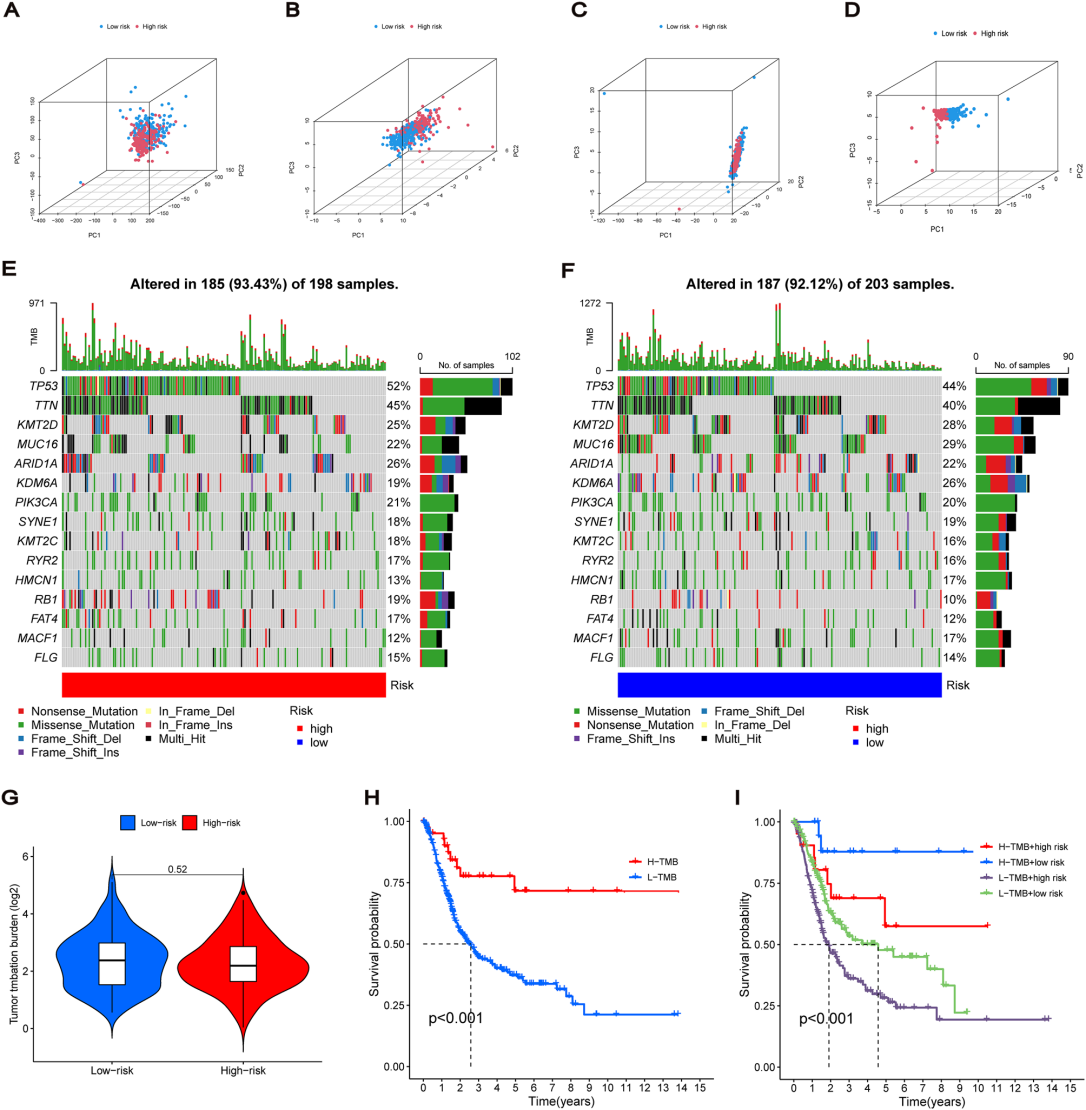
Principal Component Analysis and Tumor Mutation Burden. (**A**–**D**) 3D scatterplot of sample distribution between the low-risk and high-risk BC patients based on the entire gene expression profile, cuproptosis genes, cuproptosis-related lncRNAs, and the lncRNA profile of risk signature, respectively. (**E**,**F**) Waterfall diagram containing the top 15 genes with high mutation frequency in the low-risk and high-risk groups. (**G**) Tumor mutation burden for two groups. (**H**) Kaplan–Meier curves of OS for patients in high and low TMB subgroups. (**I**) Kaplan–Meier curve of OS for patients classified by TMB and risk signature.

We used PCA to compare the differential expression distribution between the low- and high-risk groups of the risk model, including the entire gene expression profile, cuproptosis genes, cuproptosis-related lncRNAs and the lncRNA profile of the risk model. PCA results revealed that the risk model grouping could well distinguish between low- and high-risk groups, with obvious aggregation signatures (Fig. 7A–D). Then, somatic mutations in the low- and high-risk groups were analysed, and a waterfall plot containing the top 15 genes with high mutation frequency was drawn (Fig. 7E,F). There was no significant difference in tumour mutation burden (TMB) between the two groups (Fig. 7G). According to the TMB score, each BC sample could be divided into high- and low-mutation groups. Survival analysis showed that the low-mutation group had a lower survival rate than the high-mutation group. Additionally, the low-mutation + high-risk group had the worst prognosis (Fig. 7H,I).

### Functional enrichment of cuproptosis-related lncRNAs

**Figure 8:**
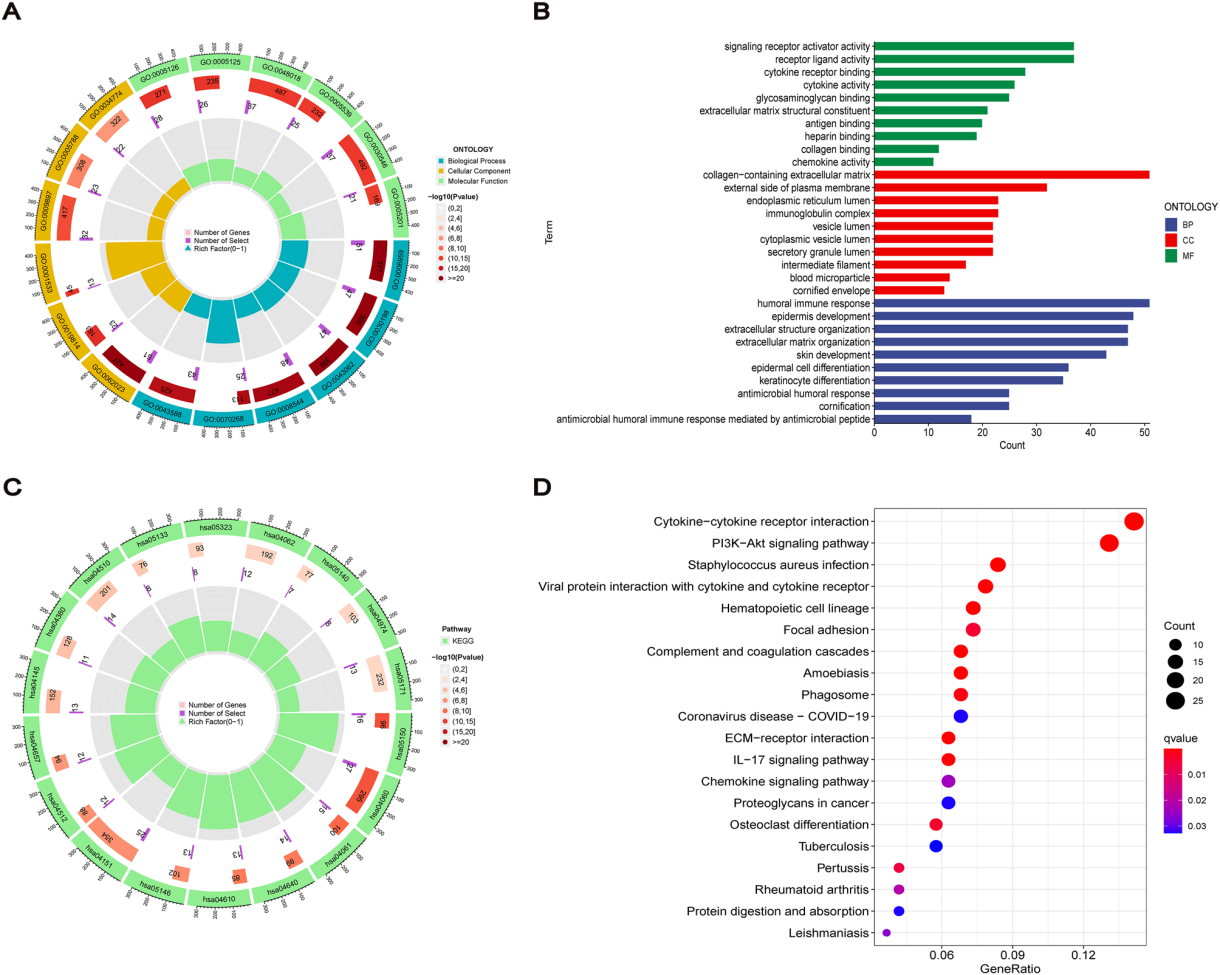
Functional enrichment of cuproptosis-related lncRNAs with risk signature. (**A**) Circos plot for GO enrichment. (**B**) Barplot for GO enrichment. (**C**) Circos plot for KEGG pathways. (**D**) Bubble graph for KEGG pathways.

We performed GO and KEGG enrichment analyses to distinguish biological differences among different risk groups. GO results showed that cuproptosis-related lncRNAs were associated with signal transduction and cellular composition and involved in multiple immune-related molecular functions and pathways, mainly humoral immune response, cytokine receptor binding and cytokine activation (Fig. 8A,B). KEGG pathway analysis showed that cuproptosis-related lncRNAs were highly correlated with the PI3K−Akt signalling pathway and were enriched in immune-related pathways such as cytokine receptor interaction and viral protein and cytokine–cytokine receptor interaction (Fig. 8C,D).

### Tumour immune microenvironment for risk signature

**Figure 9:**
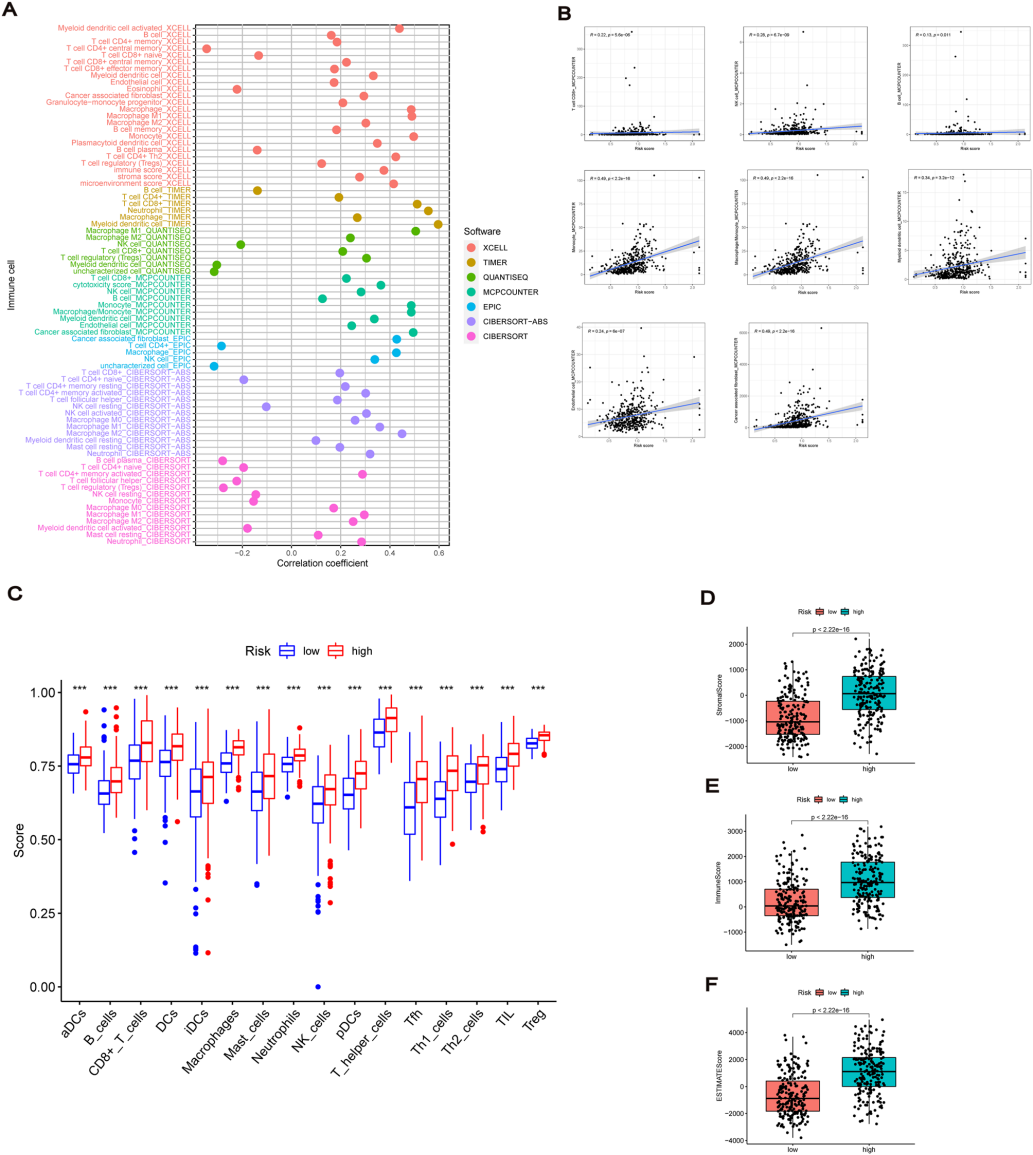
Tumor immune microenvironment for risk signature. (**A**) The immune cell bubble plot showing the abundance of immune cell infiltrates in seven algorithms. (**B**) The correlation results between risk score and immune cell subsets in MCPCOUNTER platform. (**C**) SsGSEA scores in 16 types of immune cells between the two risk subgroups. (**D**–**F**) The boxplots of StromalScore, ImmuneScore, and ESTIMATEScore in low–risk and high-risk groups. *P < 0.05, **P < 0.01, ***P < 0.001.

Based on GO and KEGG enrichment results, we explored the relationship of the risk model with immunology. Seven algorithms were used to evaluate the abundance of immune cell infiltrates in the TCGA cohort. The immune cell bubble plot showed that more immune cells were associated with patients in the high-risk group than in the low-risk group (Fig. 9A). Furthermore, the higher the risk score of the sample, the stronger the correlation with most immune cells. MCPCOUNTER analysis of the correlation results between risk score and immune cell subsets are shown in Fig. 9B. The enrichment of immune cell subsets was further quantified using ssGSEA, wherein the high-risk group had high levels of infiltration in all immune cell subsets compared with the low-risk group (Fig. 9C). Additionally, the immune scores of high-risk patients were significantly higher than those of the low-risk group, including the StromalScore, ImmuneScore and ESTIMATEScore values (Fig. 9D–F).

**Figure 10:**
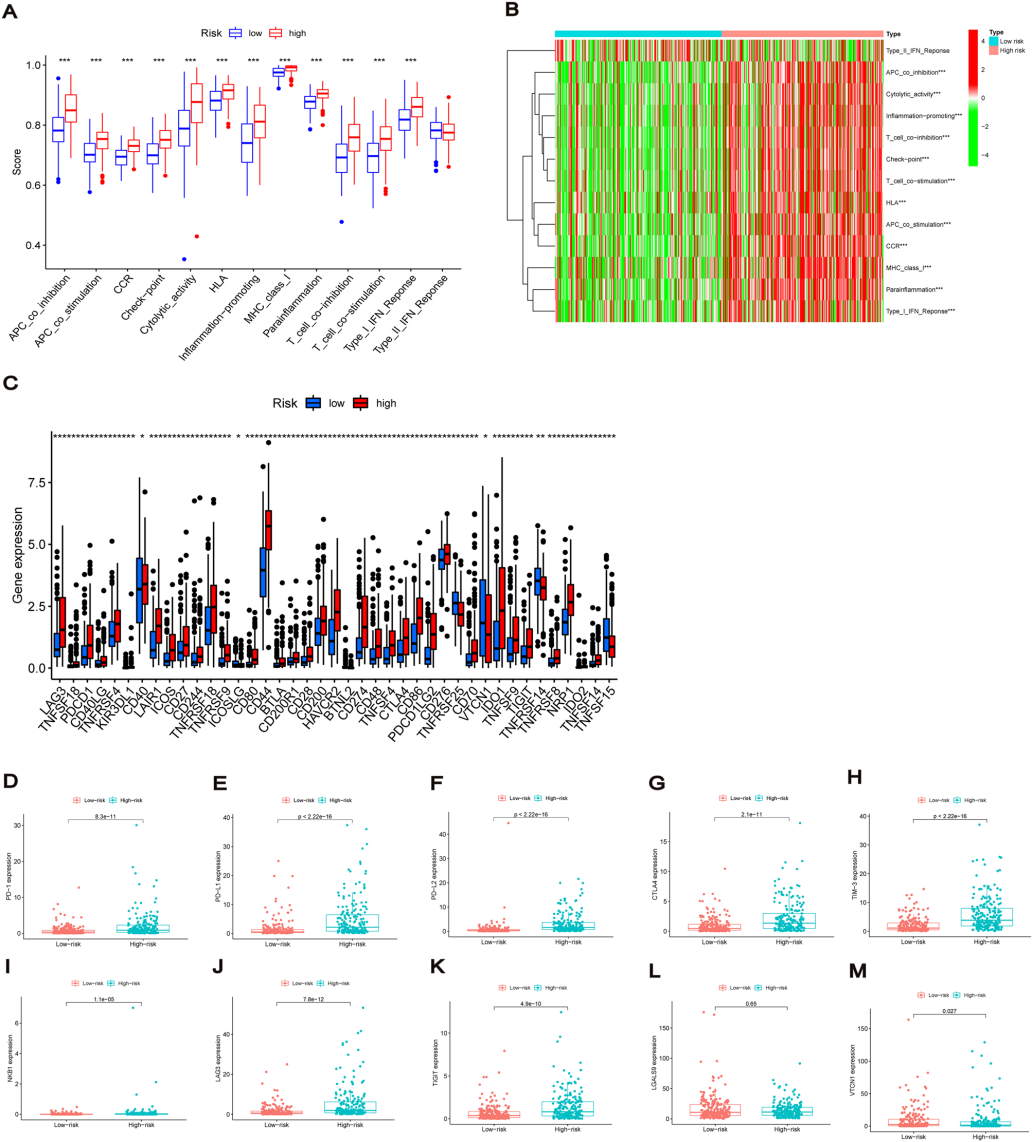
Immune-related pathways and immune checkpoints for risk signature. (**A**) SsGSEA scores for immune pathways between the two risk subgroups. ns represent not significant. (**B**) The heatmap of immune pathways between the two risk subgroups. (**C**) The comparison of immune checkpoint activation between the different risk groups. (**D**–**M**) Differential expression of common immune checkpoints (PD-1, PD-L1, PD-L2, CTLA-4, TIM-3, NKB1, LAG3, TIGIT, LGALS9, and VTCN1) between the low-risk and high-risk patients. *P < 0.05, **P < 0.01, ***P < 0.001.

Next, we explored the association of risk scores with the activation of immune pathways. Furthermore, the boxplot of ssGSEA showed significant differences in most immune pathways between the two risk subgroups except for type II interferon response (Fig. 10A). The high-risk group accompanied the activation of various immune pathways, and the heatmap of immune pathways showed that the high-risk group was closely associated with common immune pathways (Fig. 10B). Furthermore, on comparing the immune checkpoint activation between the different risk groups, a majority of the immune checkpoint-related genes were highly expressed in the high-risk group (Fig. 10C). Several common immune checkpoints showed higher activity in the high-risk group, including PD-1, PD-L1, PD-L2, CTLA-4, TIM-3, NKB1, LAG3, TIGIT, LGALS9 and VTCN1 (Fig. 10D–M). These results suggest that patients with BC in the high-risk group have a more active immune function and could be more sensitive to immunotherapy.

### Immunotherapy for risk signature and prediction of potential drugs

**Figure 11:**
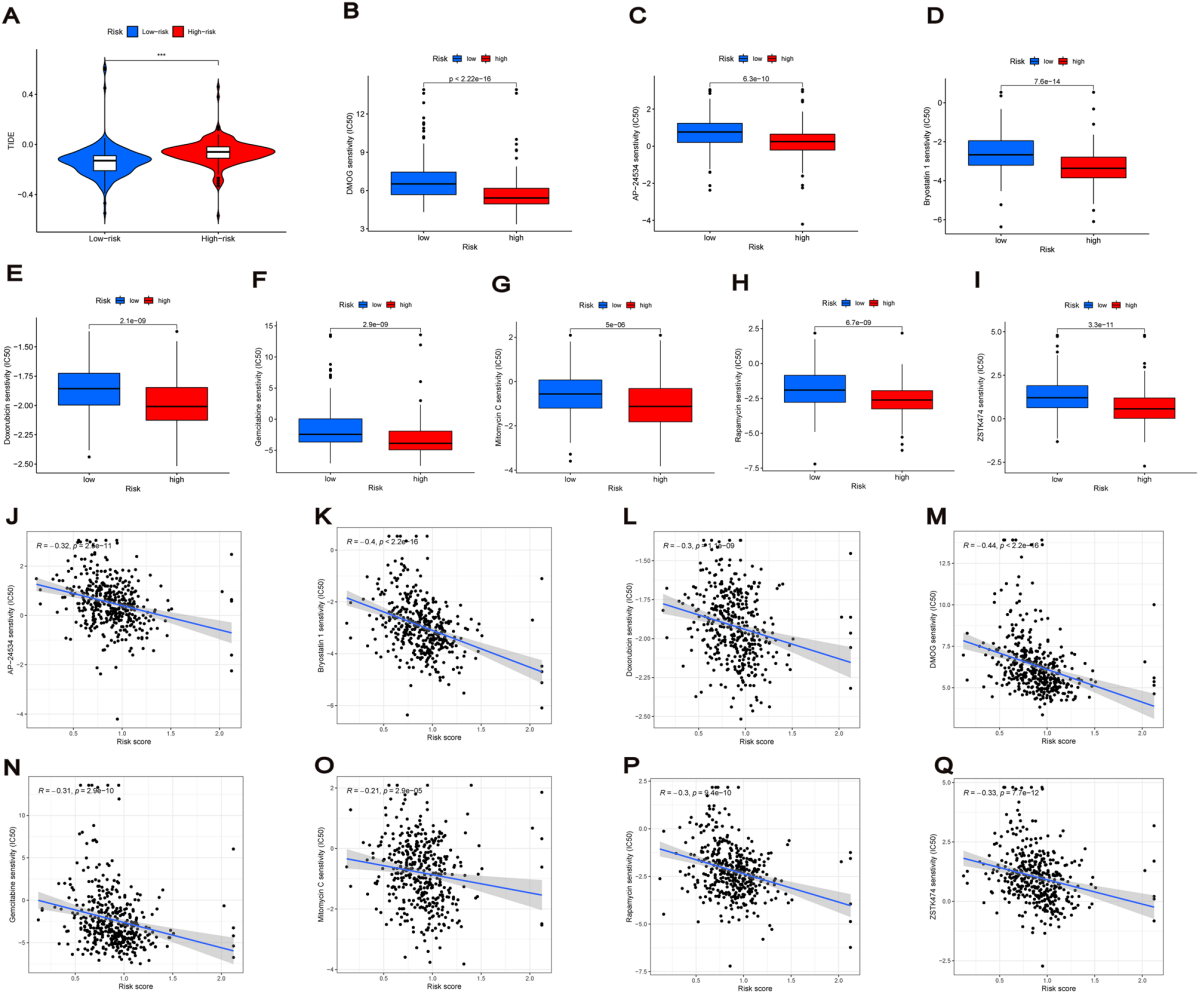
Immunotherapy for risk signature and prediction of potential drugs. (**A**) TIDE score for predicting the response of immune checkpoint inhibition therapy in different risk groups. (**B**–**I**) IC50 of eight drugs (AP-24534, Bryostatin 1, DMOG, Doxorubicin, Gemcitabine, Mitomycin C, Rapamycin and ZSTK474) differed for BC patients in different risk groups. (**J**–**Q**) The correlation of risk scores with IC50 of eight drugs.

TIDE results indicated that the high-risk group could have better efficacy for immunotherapy (Fig. 11A), suggesting that appropriate checkpoint inhibitors for patients with BC having different risks can be selected. Then, by predicting potential treatments, we found that the IC50 of eight drugs (AP-24534, Bryostatin 1, DMOG, Doxorubicin, Gemcitabine, Mitomycin C, Rapamycin and ZSTK474) differed in the risk groups. The high-risk group was more sensitive to these potential therapeutic agents (Fig. 11B–I), and risk scores were significantly correlated with the IC50 of the drugs (Fig. 11J–Q). Thus, this risk signature can aid in personalised treatments.

## Discussions

BC has serious effects on human health and life and is accompanied by high treatment costs^1,38^. Recently, many lncRNAs have been found to be involved in the occurrence, progression and metastasis of BC. lnc00892 has been reported to inhibit BC metastasis by suppressing nucleolin-mediated RhoA/RhoC mRNA stabilization^39^. Given the critical role of lncRNAs in BC, various similar phenotypic lncRNAs have been suggested as potential biomarkers to predict the prognosis of patients with BC^40,41^. However, the signatures and roles of cuproptosis-related lncRNAs remain unexplored. In this study, we constructed a signature model of lncRNAs associated with cuproptosis.

We identified 19 genes associated with cuproptosis from published literature. A total of 762 cuproptosis-related lncRNAs were obtained using co-expression analysis, and 27 prognostic lncRNAs were identified using univariate Cox analysis. Consensus cluster analysis divided BC samples into two clusters, with Cluster 1 having a poorer prognosis than Cluster 2. The expression levels of immune checkpoints such as PD-1, PD-L1, CTLA-4 and TIM-3 were also significantly higher in Cluster 1 and had a significant correlation with prognostic-related lncRNAs. Furthermore, consensus clustering suggested that Cluster 1 had a worse prognosis and could be more sensitive to immunotherapy. LASSO regression was performed on prognosis-related lncRNAs, and seven lncRNAs significantly associated with OS (AC073534.2, AC021321.1, HYI-AS1, PPP1R26-AS1, AC010328.1, AC012568.1 and MIR4435-2HG) were identified to establish the cuproptosis-associated lncRNAs signature. PPP1R26-AS1 has been reported to be upregulated in breast cancer tissues and associated with shorter OS. Moreover, PPP1R26-AS1has been reported to be a specific biomarker for the identification of luminal B subtype^42,43^. In acute myeloid leukaemia, AC073534.2 has been associated with clinical prognosis, and risk models constructed with several other prognostically relevant lncRNAs predict survival in patients with AML^44^. MIR4435-2HG acts as an oncogene and plays an important role in many cancers^45^. Expression of MIR4435-2HG is generally upregulated in gastric cancer, hepatocellular carcinoma, and ovarian cancer, and involved in biological functions through ceRNA network^46–48^. This study proposed for the first time that the AC021321.1, HYI-AS1, AC010328.1 and AC012568.1 lncRNAs could be associated with the prognosis of BC. Although their roles in various cancer remain unknown, these newly identified lncRNAs contribute to a better understanding of the pathogenic mechanisms of BC, which needs further exploration.

To assess the prognostic value of cuproptosis-associated lncRNA signatures in different risk subgroups, patients with BC were divided into high- and low-risk groups based on risk scores. The high-risk group had worse outcomes, and the risk score was an independent predictor of prognosis. The ROC curve also showed that the established lncRNA signatures had good prognostic value for BC. Kaplan–Meier analysis of different clinicopathological stratification showed that the feature model could predict the prognosis of patients with BC independently of the clinicopathological parameters. Moreover, the calibration curves confirmed a good agreement between the predicted and actual survival rates of the established nomogram for 1, 3 and 5 years. A comprehensive analysis of the established signature model revealed its accurate predictive ability for the prognosis of patients with BC.

Furthermore, pathway enrichment analysis showed that cuproptosis-related lncRNAs were highly correlated with the PI3K−Akt signalling pathway. More than half of the patients with BC from the TCGA cohort had altered the PI3K pathway, and the suppression of PI3K expression inhibited the growth and migration of cancer cells. The combination of PI3K inhibitor and anti-PD-1 therapy improved the anti-tumour effect by improving the immune-stimulating tumour microenvironment^49^. Moreover, lncRNA ADAMTS9-AS1 promoted the invasion and migration of BC cells through the activation of the PI3K/AKT/mTOR signalling pathway^50^.

Notably, the cuproptosis-associated lncRNA signature model was strongly associated with tumour immune invasion. Immune cell bubble plots based on different platforms showed that patients in the high-risk group were associated with a higher abundance of immune cell infiltrates. ssGSEA further revealed that the high-risk group had significantly high levels of infiltration in all immune cell subsets. Except for type II interferon responses, there were significant differences in most immune pathways between the two risk subgroups, with the activation of numerous immune pathways in the high-risk group. The comparison of immune checkpoint activation between the different risk groups revealed that almost all immune checkpoint genes were highly activated in the high-risk group. Studies have reported that the expression level of immune checkpoint genes was highly correlated with the efficacy of immunotherapy^51^. Moreover, TIDE analysis suggested that the high-risk group was more responsive to immunotherapy, highlighting the fact that patients at a higher risk are more likely to benefit from immune checkpoint blockade immunotherapy. Currently, nivolumab and atezolizumab, as the representative PD-1 and PD-L1 inhibitors, respectively, have been proven to benefit patients with high-risk or metastatic BC^52,53^. Finally, we identified eight drugs that had better efficacy in the high-risk group for patients with BC. These findings could aid in drug screening and clinical treatment decisions.

Although we confirmed the validity of our feature model from multiple perspectives, there remain some limitations in our study. First, we used data obtained from an online database (TCGA). However, microarray data from other sources are biased, for example, Gene Expression Omnibus data lacks complete lncRNA information. Therefore, it was difficult to conduct external validation using other clinical databases. Second, in vitro and in vivo experiments could not be conducted to further verify differences in molecular expression levels and biological functions. However, clinical samples and reliable clinical data sets for external validation are required in the future.

## Conclusions

Cuproptosis-associated lncRNA feature models can predict survival in patients with BC and could have value as independent prognostic factors. Moreover, we systematically analysed the relationship between cuproptosis-associated lncRNA signatures, TMB, tumour microenvironment and immune cell infiltration and predicted potential therapeutic drugs. To the best of our knowledge, this is the first study to elucidate potential cuproptosis-related lncRNAs that could guide personalised treatments of patients with BC.

## Supplementary Information

Supplementary Table S1.

Supplementary Table S2.

## Data Availability

All data in this study are available in online repositories. The name of the repository or datasets can be found in the article and the code for processing the data can be obtained from the corresponding author by reasonable request.
