# Supplementary material for: Identification of cuproptosis-related long noncoding RNA signature for predicting prognosis and immunotherapy response in bladder cancer
Source: Sci Rep. 2022 Dec 10;12:21386. doi: 10.1038/s41598-022-25998-2 (PMC9741610; doi:10.1038/s41598-022-25998-2)
Supplement: Supplementary file 1 — Supplementary Table S1. [file 41598_2022_25998_MOESM1_ESM.pdf]

Table S1. 19 Cuproptosis-Related Genes.

Cuproptosis-Related Genes	Name
NFE2L2	Nuclear factor erythroid 2-related factor 2
NLRP3	NLR family pyrin domain containing 3
ATP7B	Copper-transporting ATPase 2
ATP7A	Copper-transporting ATPase 1
SLC31A1	Solute carrier family 31 member 1
FDX1	Ferredoxin 1
LIAS	Lipoyl synthase
LIPT1	Lipoyltransferase 1
LIPT2	Lipoyltransferase 2
DLD	Dihydrolipoyl dehydrogenase
DLAT	Dihydrolipoamide acetyltransferase
PDHA1	Pyruvate dehydrogenase E1 subunit alpha 1
PDHB	Pyruvate dehydrogenase E1 subunit beta
MTF1	Metal regulatory transcription factor 1
GLS	Glutaminase
CDKN2A	Cyclin-dependent kinase inhibitor 2A
DBT	Dihydrolipoamide branched chain transacylase E2
GCSH	Glycine cleavage system H protein
DLST	Dihydrolipoamide succinyltransferase
