# Supplementary material for: Identification of cuproptosis-related long noncoding RNA signature for predicting prognosis and immunotherapy response in bladder cancer
Source: Sci Rep. 2022 Dec 10;12:21386. doi: 10.1038/s41598-022-25998-2 (PMC9741610; doi:10.1038/s41598-022-25998-2)
Supplement: Supplementary file 2 — Supplementary Table S2. [file 41598_2022_25998_MOESM2_ESM.pdf]

Table S2. 762 Cuproptosis-Related LncRNAs.

Cuproptosis Genes	lncRNA	Correlation	p-value	Regulation
CDKN2A	CDKN2B-AS1	0.432548	3.26E-20	postive
ATP7A	AC127024.3	0.413214	2.02E-18	postive
DBT	AC022893.1	0.406097	8.64E-18	postive
ATP7A	NCBP2-AS1	0.483678	1.52E-25	postive
DBT	NCBP2-AS1	0.480105	3.83E-25	postive
LIAS	C8orf44	0.402364	1.83E-17	postive
DBT	AL512343.2	0.461016	4.52E-23	postive
ATP7A	AL359921.1	0.427111	1.07E-19	postive
DBT	AC073534.2	0.528384	5.28E-31	postive
DBT	AC011472.4	0.573388	2.20E-37	postive
NLRP3	LINC01150	0.45904	7.28E-23	postive
PDHA1	DM1-AS	0.450736	5.22E-22	postive
ATP7A	NR2F2-AS1	0.434647	2.05E-20	postive
DBT	NR2F2-AS1	0.479205	4.83E-25	postive
ATP7A	AL590729.1	0.502572	9.37E-28	postive
DBT	AL590729.1	0.459178	7.04E-23	postive
ATP7A	AC087286.2	0.460206	5.50E-23	postive
ATP7A	AP005057.1	0.436634	1.31E-20	postive
DBT	AP005057.1	0.494562	8.42E-27	postive
LIAS	AL391684.1	0.454719	2.04E-22	postive
DBT	AL391684.1	0.469961	5.02E-24	postive
ATP7A	AC010976.1	0.469516	5.61E-24	postive
DBT	AC010976.1	0.493041	1.27E-26	postive
ATP7A	AL353804.1	0.49438	8.84E-27	postive
ATP7A	AC005972.3	0.424111	2.04E-19	postive
DBT	AC005972.3	0.592287	2.28E-40	postive
ATP7A	AC134407.1	0.465452	1.53E-23	postive
DBT	AC134407.1	0.423486	2.33E-19	postive
ATP7A	AC020978.1	0.451664	4.20E-22	postive
ATP7A	AC023483.1	0.437837	1.00E-20	postive
DBT	AC026782.2	0.410092	3.84E-18	postive
ATP7A	AC010132.4	0.475434	1.27E-24	postive
DBT	AC010132.4	0.491858	1.74E-26	postive
DBT	PDXDC2P-NPI	0.439114	7.54E-21	postive
ATP7A	AC016727.3	0.425683	1.45E-19	postive
ATP7A	AC024075.1	0.502546	9.44E-28	postive
ATP7A	MKNK1-AS1	0.482021	2.33E-25	postive
ATP7A	AC066613.1	0.51871	9.40E-30	postive
ATP7A	LINC02569	0.419452	5.49E-19	postive
DBT	LINC02569	0.575931	8.96E-38	postive
ATP7A	SPAG5-AS1	0.455869	1.56E-22	postive
DBT	SPAG5-AS1	0.566165	2.71E-36	postive
ATP7A	NDUFA6-DT	0.438566	8.53E-21	postive
ATP7A	AC068790.7	0.471678	3.26E-24	postive
ATP7A	AL109923.1	0.442817	3.26E-21	postive
DBT	AL109923.1	0.459705	6.20E-23	postive
ATP7A	AL021368.2	0.483076	1.77E-25	postive
LIAS	AL021368.2	0.405269	1.02E-17	postive
DBT	AL021368.2	0.452155	3.75E-22	postive
ATP7A	AC022973.4	0.493498	1.12E-26	postive
DBT	AC022973.4	0.438136	9.39E-21	postive
ATP7A	AL031666.2	0.430348	5.28E-20	postive
DBT	AL031666.2	0.483438	1.61E-25	postive
ATP7A	AL109614.1	0.402743	1.69E-17	postive
ATP7A	AC087286.4	0.461848	3.69E-23	postive
GCSH	AC233280.1	0.446705	1.33E-21	postive

ATP7A	ZDHHC20-IT1	0.486344	7.54E-26	postive
DBT	ZDHHC20-IT1	0.504332	5.74E-28	postive
ATP7A	AP000873.2	0.438211	9.23E-21	postive
ATP7A	AL022311.1	0.433614	2.57E-20	postive
GCSH	AL022311.1	0.449545	6.90E-22	postive
NLRP3	SOCAR	0.415843	1.17E-18	postive
DBT	AC004882.3	0.559081	3.00E-35	postive
ATP7A	AL121989.1	0.466359	1.23E-23	postive
ATP7A	PRR26	0.424904	1.72E-19	postive
ATP7A	AC087276.2	0.506686	2.97E-28	postive
ATP7A	LINC01876	0.425483	1.52E-19	postive
DBT	LINC01876	0.54999	6.03E-34	postive
DBT	LINC02289	0.537068	3.68E-32	postive
ATP7A	AC245884.10	0.425623	1.47E-19	postive
DBT	AC245884.10	0.434527	2.10E-20	postive
GCSH	AC141002.1	0.422145	3.10E-19	postive
ATP7A	PABPC4-AS1	0.492668	1.40E-26	postive
ATP7A	AL732509.1	0.487147	6.10E-26	postive
DBT	AL732509.1	0.518632	9.62E-30	postive
ATP7A	AC092301.1	0.443488	2.79E-21	postive
ATP7A	LINC00641	0.410364	3.63E-18	postive
ATP7A	YEATS2-AS1	0.423341	2.40E-19	postive
ATP7B	AC011453.1	0.426033	1.35E-19	postive
ATP7A	TRAF3IP2-AS1	0.40172	2.07E-17	postive
ATP7A	MCCC1-AS1	0.400551	2.62E-17	postive
DBT	AC021321.1	0.441859	4.05E-21	postive
DBT	LINC01376	0.406923	7.31E-18	postive
ATP7A	AC068481.1	0.419831	5.07E-19	postive
ATP7A	AL359762.3	0.513933	3.77E-29	postive
DBT	AL359762.3	0.434646	2.05E-20	postive
GCSH	AL359762.3	0.400513	2.64E-17	postive
ATP7A	AC092611.1	0.431183	4.40E-20	postive
DBT	AC092611.1	0.498183	3.14E-27	postive
ATP7A	AC092279.1	0.460914	4.63E-23	postive
DBT	AC092279.1	0.578822	3.19E-38	postive
NLRP3	MAP3K20-AS1	0.412848	2.18E-18	postive
ATP7A	MIR2052HG	0.400545	2.62E-17	postive
DBT	MIR2052HG	0.589806	5.77E-40	postive
DBT	ZNF433-AS1	0.420658	4.25E-19	postive
ATP7A	AL365436.2	0.41619	1.09E-18	postive
DBT	AL445231.1	0.466265	1.25E-23	postive
ATP7A	AC008434.1	0.421118	3.86E-19	postive
ATP7A	SSBP3-AS1	0.469899	5.09E-24	postive
ATP7A	PCAT7	0.400262	2.77E-17	postive
DBT	PCAT7	0.425433	1.53E-19	postive
ATP7A	LINC-PINT	0.430012	5.68E-20	postive
DBT	AC106771.1	0.437432	1.10E-20	postive
ATP7A	AC022150.4	0.488998	3.74E-26	postive
DBT	AC022150.4	0.607479	6.48E-43	postive
NLRP3	GAPLINC	0.46623	1.26E-23	postive
ATP7A	AL035411.3	0.563371	7.05E-36	postive
DBT	AL035411.3	0.501524	1.25E-27	postive
NLRP3	AL596223.1	0.427696	9.41E-20	postive
ATP7A	ODF2-AS1	0.464947	1.73E-23	postive
ATP7A	AP000692.1	0.459945	5.85E-23	postive
ATP7A	AC005104.1	0.433415	2.69E-20	postive
NLRP3	AL133371.2	0.569944	7.35E-37	postive
GCSH	AL162741.1	0.413469	1.92E-18	postive

DBT	AC078983.1	0.491057	2.16E-26	postive
DBT	AC253576.2	0.426796	1.14E-19	postive
ATP7A	AL133243.1	0.432175	3.53E-20	postive
GCSH	AC137932.3	0.426999	1.09E-19	postive
DBT	AL138701.2	0.567067	1.99E-36	postive
ATP7A	AL162727.2	0.485801	8.70E-26	postive
DBT	AL162727.2	0.428911	7.22E-20	postive
DBT	AC067750.1	0.46753	9.17E-24	postive
ATP7A	AC004943.1	0.419872	5.02E-19	postive
ATP7A	AL121652.1	0.413679	1.83E-18	postive
DBT	AL121652.1	0.566322	2.57E-36	postive
ATP7A	LINC02163	0.400842	2.47E-17	postive
DBT	LINC02163	0.46299	2.80E-23	postive
ATP7A	AC074194.1	0.457699	1.00E-22	postive
ATP7A	AC022211.1	0.419709	5.20E-19	postive
DBT	AC132807.2	0.40362	1.42E-17	postive
ATP7A	CDC42-IT1	0.472452	2.69E-24	postive
DBT	CDC42-IT1	0.473432	2.10E-24	postive
ATP7A	WASHC5-AS1	0.50826	1.90E-28	postive
DBT	WASHC5-AS1	0.515712	2.25E-29	postive
DBT	AL157400.4	0.446864	1.29E-21	postive
DBT	FIRRE	0.498725	2.71E-27	postive
DBT	AL645608.1	0.414788	1.46E-18	postive
ATP7A	SDK1-AS1	0.454696	2.06E-22	postive
DBT	SDK1-AS1	0.570849	5.36E-37	postive
ATP7A	AC007619.1	0.457387	1.08E-22	postive
ATP7A	AC025442.2	0.451991	3.89E-22	postive
DBT	AC025442.2	0.4056	9.55E-18	postive
ATP7A	AC087294.1	0.483711	1.50E-25	postive
DBT	AC087294.1	0.462448	3.19E-23	postive
ATP7A	AC108449.2	0.579688	2.34E-38	postive
DBT	AC108449.2	0.476559	9.51E-25	postive
ATP7A	AC073140.1	0.435517	1.69E-20	postive
ATP7A	AC138956.2	0.523692	2.16E-30	postive
DBT	AC138956.2	0.442287	3.67E-21	postive
ATP7A	FBXO30-DT	0.424442	1.90E-19	postive
ATP7A	AC078778.1	0.483534	1.57E-25	postive
DBT	AC078778.1	0.452942	3.11E-22	postive
ATP7A	PAXIP1-AS2	0.548629	9.38E-34	postive
DBT	PAXIP1-AS2	0.422141	3.10E-19	postive
DBT	AC099482.1	0.601033	8.10E-42	postive
ATP7A	AP001001.1	0.504764	5.09E-28	postive
ATP7A	AC087286.1	0.480528	3.43E-25	postive
ATP7A	AC110769.2	0.46574	1.43E-23	postive
DBT	AC110769.2	0.438693	8.29E-21	postive
ATP7A	AC129510.1	0.402432	1.80E-17	postive
ATP7A	USP3-AS1	0.444286	2.33E-21	postive
ATP7A	AC007620.3	0.4269	1.12E-19	postive
DBT	AC009318.3	0.590583	4.32E-40	postive
ATP7A	AL353804.2	0.480146	3.79E-25	postive
GCSH	AL353804.2	0.401155	2.32E-17	postive
ATP7A	SAP30L-AS1	0.514643	3.07E-29	postive
ATP7A	AP001178.1	0.419961	4.93E-19	postive
DBT	AP001178.1	0.461468	4.05E-23	postive
ATP7A	AC068533.3	0.453532	2.71E-22	postive
DBT	AC068533.3	0.402881	1.65E-17	postive
ATP7A	MAL2-AS1	0.411463	2.90E-18	postive
DBT	MAL2-AS1	0.57257	2.94E-37	postive

ATP7A	LINC00630	0.573316	2.26E-37	postive
DBT	LINC00630	0.452925	3.12E-22	postive
ATP7A	Z82243.1	0.457582	1.03E-22	postive
GCSH	Z82243.1	0.40804	5.83E-18	postive
DBT	HYI-AS1	0.46369	2.36E-23	postive
ATP7A	DCUN1D2-AS	0.460481	5.14E-23	postive
LIAS	AC107068.1	0.464982	1.72E-23	postive
ATP7A	AC006270.1	0.403453	1.47E-17	postive
DBT	AC006270.1	0.546737	1.73E-33	postive
ATP7A	AC020978.2	0.463685	2.36E-23	postive
ATP7A	AC005014.4	0.520675	5.28E-30	postive
DBT	AC005014.4	0.49272	1.38E-26	postive
ATP7A	AC018521.6	0.417537	8.21E-19	postive
DBT	AC018521.6	0.563952	5.78E-36	postive
ATP7A	AC011477.2	0.455392	1.74E-22	postive
ATP7A	AL445309.1	0.442812	3.26E-21	postive
DBT	AL445309.1	0.474898	1.45E-24	postive
ATP7A	AP000487.2	0.408069	5.79E-18	postive
DBT	HDAC2-AS2	0.409535	4.30E-18	postive
ATP7A	AL390719.3	0.487736	5.22E-26	postive
ATP7A	AL138921.1	0.436441	1.37E-20	postive
DBT	AL138921.1	0.493726	1.05E-26	postive
LIAS	UBA6-AS1	0.415378	1.29E-18	postive
DBT	LINC02340	0.435159	1.83E-20	postive
ATP7A	AC073896.3	0.410178	3.77E-18	postive
ATP7A	AC022137.3	0.484321	1.28E-25	postive
DBT	AC022137.3	0.45971	6.20E-23	postive
ATP7A	AC023794.4	0.439575	6.79E-21	postive
DBT	AC023794.4	0.52262	2.97E-30	postive
CDKN2A	AL449423.1	0.578029	4.24E-38	postive
ATP7A	AL353697.1	0.441261	4.64E-21	postive
DBT	AL353697.1	0.490693	2.38E-26	postive
DBT	AL929236.1	0.406601	7.80E-18	postive
ATP7A	AC138207.7	0.444976	1.99E-21	postive
DBT	AC004233.1	0.423733	2.21E-19	postive
ATP7A	ZNF32-AS2	0.447232	1.18E-21	postive
ATP7A	AC007681.1	0.540216	1.37E-32	postive
DBT	AC007681.1	0.535721	5.59E-32	postive
DBT	AC027243.2	0.51694	1.58E-29	postive
GCSH	AC073389.1	0.462093	3.48E-23	postive
DBT	AC018616.1	0.610686	1.80E-43	postive
LIPT1	AC012360.3	0.431199	4.38E-20	postive
PDHA1	FP671120.7	0.435679	1.63E-20	postive
ATP7A	AC012459.1	0.481283	2.82E-25	postive
DBT	AC012459.1	0.452457	3.49E-22	postive
ATP7A	AC015849.3	0.473811	1.91E-24	postive
DBT	AC108066.2	0.568868	1.07E-36	postive
ATP7A	AC024270.3	0.455007	1.91E-22	postive
ATP7A	AC004884.2	0.467176	1.00E-23	postive
DBT	AC004884.2	0.408999	4.79E-18	postive
ATP7A	AC011933.3	0.420844	4.09E-19	postive
ATP7A	LINC01126	0.430331	5.30E-20	postive
DBT	LINC01126	0.485202	1.02E-25	postive
NLRP3	AC131097.3	0.491199	2.08E-26	postive
DBT	LIPC-AS1	0.568521	1.20E-36	postive
ATP7A	AC244093.5	0.521751	3.84E-30	postive
ATP7A	AC007528.1	0.47624	1.03E-24	postive
DBT	AC007528.1	0.451392	4.48E-22	postive

NLRP3	AC007877.1	0.49417	9.36E-27	postive
ATP7A	AC007128.1	0.469084	6.24E-24	postive
DBT	AC007128.1	0.503371	7.51E-28	postive
ATP7A	RUFY1-AS1	0.436729	1.29E-20	postive
ATP7A	AC008115.3	0.516817	1.63E-29	postive
ATP7A	AC024267.5	0.511075	8.55E-29	postive
DBT	AC024267.5	0.423172	2.49E-19	postive
ATP7A	AL133445.2	0.41251	2.33E-18	postive
ATP7A	AC005522.1	0.456839	1.23E-22	postive
DBT	AC005522.1	0.444136	2.41E-21	postive
ATP7A	AC092794.1	0.425613	1.48E-19	postive
ATP7A	IQCJ-SCHIP1-/	0.43017	5.49E-20	postive
ATP7A	RAP2C-AS1	0.495813	6.00E-27	postive
ATP7A	AC005253.1	0.425468	1.52E-19	postive
CDKN2A	CDKN2A-DT	0.804384	9.43E-95	postive
ATP7A	AC068790.6	0.44646	1.41E-21	postive
ATP7A	AC092802.2	0.547452	1.37E-33	postive
DBT	AC092802.2	0.5555	9.89E-35	postive
ATP7A	AC011405.1	0.533747	1.03E-31	postive
ATP7A	AC087301.1	0.404202	1.26E-17	postive
DBT	Z93403.1	0.450607	5.39E-22	postive
DBT	AC008121.2	0.519242	8.05E-30	postive
ATP7A	AC211476.2	0.441105	4.81E-21	postive
ATP7A	AC009704.2	0.447557	1.10E-21	postive
ATP7A	AC073529.1	0.530178	3.07E-31	postive
ATP7A	AL137779.1	0.430842	4.74E-20	postive
ATP7A	AL132800.1	0.486065	8.11E-26	postive
ATP7A	AC006566.1	0.40627	8.34E-18	postive
DBT	AC006566.1	0.518336	1.05E-29	postive
DBT	AC009262.1	0.577406	5.30E-38	postive
DLD	AL136981.3	0.428641	7.66E-20	postive
ATP7B	AC010328.1	0.562988	8.03E-36	postive
ATP7A	AC008543.3	0.462831	2.91E-23	postive
DBT	AC008543.3	0.489384	3.37E-26	postive
ATP7A	GLYCTK-AS1	0.442173	3.77E-21	postive
ATP7A	PCBP2-OT1	0.484081	1.36E-25	postive
DBT	PCBP2-OT1	0.412549	2.32E-18	postive
CDKN2A	LINC02765	0.407789	6.13E-18	postive
ATP7A	AP000766.1	0.530364	2.90E-31	postive
DBT	AP000766.1	0.486781	6.72E-26	postive
ATP7A	AP001107.1	0.465419	1.54E-23	postive
ATP7A	AC008870.4	0.453193	2.93E-22	postive
DBT	AC008781.2	0.546927	1.62E-33	postive
GCSH	AC007546.1	0.491688	1.82E-26	postive
ATP7A	AC058791.1	0.462396	3.23E-23	postive
ATP7A	UBOX5-AS1	0.401803	2.04E-17	postive
ATP7A	AC068790.2	0.460998	4.54E-23	postive
DBT	AC068790.2	0.433783	2.48E-20	postive
ATP7A	AC010226.1	0.436079	1.49E-20	postive
ATP7A	AC073592.1	0.435331	1.76E-20	postive
ATP7A	AC092794.2	0.417882	7.64E-19	postive
DBT	AC092794.2	0.415337	1.30E-18	postive
ATP7A	PSPC1-AS2	0.418773	6.33E-19	postive
ATP7A	AC015922.2	0.401996	1.96E-17	postive
NLRP3	AL109741.1	0.428322	8.21E-20	postive
ATP7A	AC068790.5	0.467024	1.04E-23	postive
DBT	AC108052.1	0.670856	3.42E-55	postive
DBT	LINC02466	0.400764	2.51E-17	postive

ATP7A	AC244093.4	0.440139	5.98E-21	postive
ATP7A	AC090579.1	0.415651	1.22E-18	postive
DBT	AC090579.1	0.508351	1.86E-28	postive
ATP7A	AC112484.5	0.445259	1.86E-21	postive
GCSH	SLFNL1-AS1	0.434524	2.10E-20	postive
ATP7A	AC060780.1	0.430505	5.10E-20	postive
ATP7A	AC007881.4	0.518221	1.08E-29	postive
DBT	AC007881.4	0.448067	9.73E-22	postive
ATP7A	AC023794.1	0.414038	1.70E-18	postive
DBT	AC023794.1	0.513392	4.40E-29	postive
ATP7A	AC034139.1	0.416558	1.01E-18	postive
ATP7A	AC069547.2	0.46715	1.01E-23	postive
DBT	AC069547.2	0.539977	1.48E-32	postive
ATP7A	AC008731.1	0.445482	1.77E-21	postive
ATP7A	AC146507.3	0.420221	4.66E-19	postive
ATP7A	AL110115.1	0.418393	6.86E-19	postive
ATP7A	AC006160.1	0.438842	8.01E-21	postive
GCSH	SBNO1-AS1	0.410606	3.45E-18	postive
DBT	AC063943.1	0.469457	5.69E-24	postive
ATP7A	AC008114.1	0.429653	6.14E-20	postive
DBT	AC008114.1	0.447124	1.21E-21	postive
PDHA1	AC061992.2	0.589297	6.97E-40	postive
ATP7A	FO680682.1	0.458079	9.17E-23	postive
DBT	AC093382.1	0.455316	1.77E-22	postive
DBT	LNX1-AS2	0.485084	1.05E-25	postive
DBT	NDUFB2-AS1	0.465751	1.42E-23	postive
ATP7A	AL117344.2	0.44827	9.28E-22	postive
DBT	AL117344.2	0.538391	2.44E-32	postive
ATP7A	MAP3K5-AS1	0.407785	6.14E-18	postive
DBT	MAP3K5-AS1	0.465069	1.68E-23	postive
ATP7A	AL050309.1	0.431336	4.25E-20	postive
DBT	AL050309.1	0.582405	8.77E-39	postive
DBT	AL359636.2	0.456896	1.22E-22	postive
ATP7A	COX10-AS1	0.429351	6.56E-20	postive
ATP7A	AL359878.1	0.473871	1.88E-24	postive
DBT	AL359878.1	0.574686	1.39E-37	postive
DBT	WARS2-AS1	0.495115	7.25E-27	postive
DBT	AC022165.1	0.415432	1.27E-18	postive
DBT	AC100821.2	0.419292	5.68E-19	postive
ATP7A	DLEU1	0.44837	9.07E-22	postive
DBT	DLEU1	0.578227	3.95E-38	postive
ATP7A	AC104170.1	0.440419	5.61E-21	postive
LIPT2	SOD2-OT1	0.420787	4.14E-19	postive
ATP7A	LINC00412	0.418729	6.39E-19	postive
DBT	LINC00412	0.496719	4.69E-27	postive
ATP7A	AC022272.1	0.490955	2.22E-26	postive
DBT	AC022272.1	0.560893	1.63E-35	postive
ATP7A	AC092053.3	0.417918	7.58E-19	postive
ATP7A	AC073046.1	0.442778	3.28E-21	postive
DBT	AC073046.1	0.411496	2.88E-18	postive
ATP7A	AC002128.1	0.446465	1.41E-21	postive
DBT	AC002128.1	0.493318	1.18E-26	postive
ATP7A	AP002490.1	0.410972	3.20E-18	postive
FDX1	AC007993.3	0.486242	7.74E-26	postive
ATP7A	AC016394.2	0.403769	1.38E-17	postive
DBT	AC016394.2	0.455266	1.80E-22	postive
ATP7A	AL731566.2	0.496076	5.58E-27	postive
DBT	AL731566.2	0.459606	6.35E-23	postive

NLRP3	VIM-AS1	0.428184	8.46E-20	postive
DBT	AC024361.1	0.459528	6.47E-23	postive
ATP7A	AC087854.1	0.473253	2.20E-24	postive
GCSH	ATXN2-AS	0.412961	2.13E-18	postive
GCSH	AC007448.4	0.47218	2.88E-24	postive
ATP7A	AP001628.1	0.406987	7.21E-18	postive
DBT	AP001628.1	0.510718	9.47E-29	postive
ATP7A	AC006452.1	0.431243	4.34E-20	postive
DBT	AC006452.1	0.57946	2.54E-38	postive
ATP7A	AC012557.1	0.470412	4.48E-24	postive
DBT	AC012557.1	0.507807	2.16E-28	postive
ATP7A	ANKRD10-IT1	0.483964	1.41E-25	postive
ATP7A	AP001625.2	0.45649	1.34E-22	postive
DBT	AP001625.2	0.441721	4.18E-21	postive
NLRP3	SH3RF3-AS1	0.574926	1.28E-37	postive
DBT	AC121247.1	0.401644	2.11E-17	postive
ATP7A	AC007128.2	0.50858	1.74E-28	postive
DBT	AC007128.2	0.513996	3.70E-29	postive
ATP7A	AP001432.1	0.476176	1.05E-24	postive
ATP7A	AC026803.3	0.504678	5.21E-28	postive
DBT	AC026803.3	0.560651	1.77E-35	postive
ATP7A	AC091906.1	0.430794	4.79E-20	postive
DBT	AC091906.1	0.499359	2.28E-27	postive
ATP7A	AC005070.3	0.475237	1.33E-24	postive
ATP7A	AC012568.1	0.407649	6.31E-18	postive
ATP7A	LAMC1-AS1	0.446821	1.30E-21	postive
ATP7A	AC015923.1	0.414838	1.44E-18	postive
DBT	AC015923.1	0.538176	2.60E-32	postive
GCSH	AP000593.3	0.477717	7.07E-25	postive
ATP7B	LINC01238	0.448479	8.84E-22	postive
ATP7A	AC016405.1	0.444905	2.02E-21	postive
DBT	AC016405.1	0.46964	5.43E-24	postive
DBT	AC011921.1	0.442714	3.33E-21	postive
ATP7A	AC073046.3	0.501797	1.16E-27	postive
DBT	AC073046.3	0.40203	1.95E-17	postive
ATP7A	C21orf62-AS1	0.476251	1.03E-24	postive
DBT	C21orf62-AS1	0.610561	1.90E-43	postive
ATP7A	PTOV1-AS1	0.400923	2.43E-17	postive
DBT	PTOV1-AS1	0.430053	5.63E-20	postive
ATP7A	NPTN-IT1	0.424633	1.82E-19	postive
DBT	LINC02159	0.438884	7.94E-21	postive
ATP7A	AC108463.2	0.454132	2.35E-22	postive
DBT	AC073896.2	0.513885	3.82E-29	postive
ATP7A	AC138932.4	0.476933	8.64E-25	postive
ATP7A	Z69666.1	0.469002	6.37E-24	postive
DBT	GRHL3-AS1	0.40607	8.68E-18	postive
ATP7A	N4BP2L2-IT2	0.460458	5.17E-23	postive
DBT	N4BP2L2-IT2	0.543294	5.19E-33	postive
GCSH	AC145423.1	0.430127	5.54E-20	postive
ATP7A	AC005670.3	0.411684	2.77E-18	postive
DBT	AC073283.1	0.427893	9.01E-20	postive
DBT	AC004863.1	0.428444	8.00E-20	postive
ATP7A	AL157838.1	0.467687	8.82E-24	postive
DBT	AL157838.1	0.410647	3.42E-18	postive
NLRP3	LINC02345	0.418118	7.27E-19	postive
LIPT2	AC108860.2	0.402624	1.73E-17	postive
GCSH	AC009148.1	0.436963	1.22E-20	postive
ATP7A	MORF4L2-AS1	0.428267	8.31E-20	postive

DBT	MORF4L2-AS1	0.427471	9.87E-20	postive
GCSH	AC040169.1	0.443257	2.95E-21	postive
ATP7A	AL080317.2	0.424089	2.05E-19	postive
DBT	AL080317.2	0.580236	1.92E-38	postive
GCSH	AL080317.2	0.424917	1.71E-19	postive
ATP7A	AC090589.3	0.421998	3.20E-19	postive
ATP7A	UBE2Q1-AS1	0.412033	2.58E-18	postive
NLRP3	AC090559.1	0.686796	8.91E-59	postive
ATP7A	EIPR1-IT1	0.460441	5.19E-23	postive
ATP7A	AC124283.3	0.416567	1.01E-18	postive
ATP7A	AC110611.1	0.476239	1.03E-24	postive
LIAS	AC110611.1	0.41549	1.26E-18	postive
DBT	AC110611.1	0.458953	7.44E-23	postive
ATP7A	RC3H1-IT1	0.410739	3.36E-18	postive
DBT	AC024361.3	0.520913	4.92E-30	postive
ATP7A	AP001793.1	0.473542	2.04E-24	postive
ATP7A	AC010186.3	0.474867	1.46E-24	postive
DBT	AC010186.3	0.53024	3.01E-31	postive
DBT	AL031282.2	0.44701	1.24E-21	postive
DBT	LINC02042	0.538579	2.30E-32	postive
ATP7A	AP002907.1	0.423854	2.15E-19	postive
ATP7A	AC073349.4	0.452429	3.51E-22	postive
DBT	AC073349.4	0.45898	7.39E-23	postive
DBT	AC005828.4	0.58475	3.73E-39	postive
ATP7A	AC012076.1	0.4726	2.59E-24	postive
DBT	AP005432.1	0.554454	1.40E-34	postive
NLRP3	MIR223HG	0.459438	6.62E-23	postive
ATP7A	LINC02615	0.439635	6.70E-21	postive
DBT	LINC02615	0.437737	1.03E-20	postive
DBT	AC006017.1	0.613588	5.60E-44	postive
DBT	AL355574.1	0.414272	1.62E-18	postive
ATP7A	AC114956.2	0.522852	2.77E-30	postive
DBT	AC114956.2	0.587309	1.46E-39	postive
LIAS	SCAMP1-AS1	0.400845	2.47E-17	postive
LIPT1	STARD7-AS1	0.406154	8.54E-18	postive
ATP7A	AL513327.1	0.456989	1.19E-22	postive
ATP7A	FMR1-IT1	0.450821	5.12E-22	postive
ATP7A	AF117829.1	0.401283	2.26E-17	postive
ATP7A	AL158212.5	0.48873	4.01E-26	postive
ATP7A	AP001619.1	0.502982	8.37E-28	postive
DBT	AP001619.1	0.42408	2.05E-19	postive
ATP7A	AC068790.3	0.474845	1.47E-24	postive
GCSH	AC068790.3	0.406745	7.58E-18	postive
ATP7A	LIMD1-AS1	0.495321	6.85E-27	postive
DBT	LIMD1-AS1	0.650366	6.69E-51	postive
ATP7A	AC026124.2	0.415811	1.18E-18	postive
DBT	AC026124.2	0.42984	5.90E-20	postive
GCSH	AC103769.1	0.417392	8.46E-19	postive
ATP7A	AC015849.4	0.494595	8.34E-27	postive
DBT	ZNF252P-AS1	0.410908	3.25E-18	postive
ATP7A	AC079305.2	0.418202	7.14E-19	postive
DBT	AC079305.2	0.461685	3.84E-23	postive
ATP7A	HCG25	0.469827	5.19E-24	postive
ATP7A	AC007622.2	0.508902	1.59E-28	postive
DBT	AC007622.2	0.494985	7.51E-27	postive
DBT	AC004381.1	0.410783	3.33E-18	postive
ATP7A	AC097641.2	0.430739	4.84E-20	postive
DBT	AC097641.2	0.525994	1.09E-30	postive

ATP7A	AC100763.1	0.463685	2.36E-23	postive
ATP7B	AC018755.5	0.447332	1.15E-21	postive
NLRP3	MIR222HG	0.417015	9.16E-19	postive
ATP7A	AC005856.1	0.450194	5.93E-22	postive
DBT	AC005856.1	0.499873	1.98E-27	postive
NLRP3	CYTOR	0.441043	4.87E-21	postive
LIPT2	AC091271.1	0.430408	5.21E-20	postive
NLRP3	LINC01943	0.507264	2.52E-28	postive
DBT	AP001630.1	0.579633	2.39E-38	postive
ATP7B	MIR181A2HG	0.404552	1.18E-17	postive
SLC31A1	TP53TG1	-0.40307	1.59E-17	negative
ATP7A	AC004967.2	0.504709	5.17E-28	postive
DBT	AC004967.2	0.423431	2.36E-19	postive
ATP7A	PHC2-AS1	0.413558	1.88E-18	postive
ATP7A	GUSBP11	0.404426	1.21E-17	postive
DBT	LINC02886	0.563269	7.30E-36	postive
ATP7A	AC069257.1	0.472443	2.69E-24	postive
ATP7A	AC063960.2	0.408714	5.08E-18	postive
DBT	AC063960.2	0.435184	1.82E-20	postive
ATP7A	AL513327.2	0.467219	9.91E-24	postive
GCSH	AL513327.2	0.411586	2.82E-18	postive
ATP7A	AC121764.1	0.438575	8.51E-21	postive
DBT	AC121764.1	0.554699	1.29E-34	postive
ATP7A	AC025031.2	0.49921	2.37E-27	postive
ATP7A	RNF216-IT1	0.441523	4.37E-21	postive
ATP7A	AC024267.3	0.424686	1.80E-19	postive
DBT	AC024267.3	0.457817	9.77E-23	postive
DBT	AC103746.1	0.554517	1.37E-34	postive
DBT	AC100823.1	0.540716	1.17E-32	postive
DBT	AC007255.1	0.416461	1.03E-18	postive
CDKN2A	AL441992.1	0.432761	3.11E-20	postive
ATP7A	ELOA-AS1	0.407779	6.14E-18	postive
ATP7A	AL354726.1	0.490512	2.50E-26	postive
PDHA1	AL138720.1	0.465473	1.52E-23	postive
ATP7A	PPIC-AS1	0.430775	4.81E-20	postive
DBT	AC110792.3	0.464227	2.07E-23	postive
ATP7A	AC008543.1	0.452884	3.15E-22	postive
LIAS	EPB41L4A-AS1	0.462671	3.02E-23	postive
SLC31A1	AL023284.4	-0.40066	2.56E-17	negative
ATP7A	AC009754.1	0.42312	2.52E-19	postive
DBT	AC009754.1	0.408404	5.41E-18	postive
DBT	AC010615.2	0.407198	6.91E-18	postive
DBT	AC098656.1	0.542109	7.56E-33	postive
ATP7A	CRTC3-AS1	0.520943	4.88E-30	postive
DBT	CRTC3-AS1	0.590337	4.73E-40	postive
LIAS	MIR29B2CHG	0.452585	3.38E-22	postive
DBT	MIR29B2CHG	0.435436	1.72E-20	postive
DBT	AL355388.1	0.413814	1.78E-18	postive
ATP7A	AC078846.1	0.423886	2.14E-19	postive
GCSH	AC078846.1	0.424222	1.99E-19	postive
DBT	LINC01355	0.455902	1.54E-22	postive
ATP7A	LAMTOR5-AS1	0.474998	1.41E-24	postive
DBT	LAMTOR5-AS1	0.562932	8.18E-36	postive
ATP7A	AL138756.1	0.429316	6.61E-20	postive
DBT	AL138756.1	0.561775	1.21E-35	postive
DBT	PCAT1	0.531979	1.77E-31	postive
GCSH	PCAT1	0.432126	3.57E-20	postive
DBT	AL031710.2	0.403363	1.50E-17	postive

ATP7A	AL008718.3	0.420564	4.34E-19	postive
DBT	AC007881.3	0.414234	1.63E-18	postive
ATP7A	AC124069.1	0.408503	5.30E-18	postive
DBT	AC124069.1	0.558149	4.10E-35	postive
GCSH	AL031963.3	0.448986	7.86E-22	postive
ATP7A	AC073487.1	0.479528	4.44E-25	postive
GCSH	AC145423.2	0.420366	4.52E-19	postive
ATP7A	UBL7-AS1	0.467629	8.95E-24	postive
ATP7A	AC005828.1	0.425181	1.62E-19	postive
DBT	AC005828.1	0.527803	6.30E-31	postive
DBT	AC012645.2	0.402216	1.88E-17	postive
GCSH	AC131009.3	0.44841	8.99E-22	postive
NLRP3	AC079921.2	0.48806	4.79E-26	postive
DBT	AC010300.1	0.513037	4.88E-29	postive
ATP7A	AC245884.9	0.410133	3.80E-18	postive
ATP7A	PPP1R26-AS1	0.429134	6.88E-20	postive
ATP7A	AC115102.1	0.413359	1.96E-18	postive
ATP7A	AL080317.1	0.400902	2.44E-17	postive
DBT	AL080317.1	0.586231	2.17E-39	postive
ATP7A	AC020558.6	0.425424	1.54E-19	postive
ATP7A	AL354993.2	0.50919	1.46E-28	postive
DBT	AL354993.2	0.523455	2.32E-30	postive
GCSH	AL031716.1	0.410941	3.22E-18	postive
ATP7A	AL512506.1	0.463431	2.51E-23	postive
DBT	AL512506.1	0.548002	1.15E-33	postive
DBT	MYLK-AS1	0.433885	2.42E-20	postive
ATP7A	AC025031.4	0.420387	4.50E-19	postive
DBT	AC025031.4	0.575217	1.15E-37	postive
DBT	LINC02109	0.62525	4.50E-46	postive
DBT	SH3TC2-DT	0.413829	1.78E-18	postive
ATP7A	AL354989.1	0.504692	5.19E-28	postive
ATP7A	AC106037.2	0.492684	1.40E-26	postive
DBT	AC106037.2	0.514338	3.35E-29	postive
ATP7A	AC021491.2	0.412086	2.55E-18	postive
DBT	AC021491.2	0.524814	1.55E-30	postive
ATP7A	LINC01409	0.483905	1.43E-25	postive
DBT	LINC01409	0.498819	2.64E-27	postive
ATP7A	AC018752.1	0.543198	5.35E-33	postive
DBT	AC018752.1	0.400667	2.56E-17	postive
ATP7A	AP005899.1	0.505393	4.27E-28	postive
ATP7A	AL358216.1	0.400778	2.50E-17	postive
ATP7A	AC004832.4	0.442578	3.44E-21	postive
ATP7A	AC090236.2	0.472333	2.77E-24	postive
DBT	AC090236.2	0.535767	5.51E-32	postive
LIPT2	AP001462.1	0.438621	8.42E-21	postive
ATP7A	AC099811.1	0.440372	5.67E-21	postive
DBT	LINC01322	0.401594	2.13E-17	postive
GCSH	AL136531.1	0.403755	1.38E-17	postive
ATP7A	AC005730.3	0.409525	4.31E-18	postive
DBT	U47924.1	0.48219	2.23E-25	postive
ATP7A	AC127024.2	0.401423	2.20E-17	postive
ATP7A	AL157871.5	0.472499	2.66E-24	postive
ATP7A	AC024560.4	0.43178	3.86E-20	postive
DBT	AC024560.4	0.470661	4.21E-24	postive
ATP7A	GRK5-IT1	0.448353	9.11E-22	postive
ATP7A	AC108727.1	0.457527	1.05E-22	postive
ATP7A	AC099811.5	0.477882	6.78E-25	postive
ATP7A	AC104984.2	0.468661	6.93E-24	postive

ATP7A	GNG12-AS1	0.476421	9.85E-25	postive
DBT	AC012184.3	0.408502	5.30E-18	postive
ATP7A	AC011442.1	0.43776	1.02E-20	postive
DBT	AC011442.1	0.413722	1.82E-18	postive
ATP7A	AC004637.1	0.41203	2.58E-18	postive
NLRP3	MIR4435-2HG	0.418917	6.14E-19	postive
ATP7A	AF127577.4	0.407038	7.14E-18	postive
ATP7A	AC010210.1	0.437032	1.20E-20	postive
DBT	AC010210.1	0.549589	6.87E-34	postive
ATP7A	PDXP-DT	0.409961	3.94E-18	postive
DBT	PDXP-DT	0.480751	3.24E-25	postive
LIAS	AC108471.2	0.450677	5.30E-22	postive
ATP7A	AL117327.1	0.407337	6.72E-18	postive
ATP7A	AL117329.1	0.47213	2.91E-24	postive
DBT	AL117329.1	0.45213	3.77E-22	postive
ATP7A	AC092645.2	0.444731	2.10E-21	postive
GCSH	JARID2-AS1	0.436572	1.33E-20	postive
ATP7A	SNHG14	0.422487	2.88E-19	postive
DBT	SNHG14	0.579076	2.92E-38	postive
ATP7A	GHRLOS	0.419178	5.81E-19	postive
DBT	GHRLOS	0.490147	2.75E-26	postive
ATP7A	SGMS1-AS1	0.476807	8.92E-25	postive
DBT	SGMS1-AS1	0.496706	4.70E-27	postive
DBT	AC034102.8	0.589805	5.77E-40	postive
ATP7A	AL021707.4	0.438587	8.49E-21	postive
ATP7A	AC011466.1	0.458622	8.05E-23	postive
DBT	AL163952.1	0.419254	5.72E-19	postive
ATP7A	AL133243.3	0.481955	2.37E-25	postive
ATP7A	AC005519.1	0.413067	2.08E-18	postive
ATP7A	AC127024.4	0.490758	2.34E-26	postive
GCSH	AC127024.4	0.418414	6.83E-19	postive
DBT	LINC01194	0.441969	3.95E-21	postive
LIAS	GEMIN7-AS1	0.418389	6.86E-19	postive
ATP7A	AC006480.2	0.431024	4.55E-20	postive
ATP7A	AC078795.1	0.518051	1.14E-29	postive
DBT	AC078795.1	0.417875	7.65E-19	postive
ATP7A	LINC00894	0.419912	4.98E-19	postive
DBT	LINC00894	0.430206	5.44E-20	postive
DBT	AL450344.3	0.562605	9.14E-36	postive
DBT	AC021491.4	0.567343	1.81E-36	postive
DBT	AC011503.2	0.464255	2.05E-23	postive
ATP7A	AL450384.1	0.431651	3.97E-20	postive
DBT	AL450384.1	0.564825	4.29E-36	postive
ATP7A	AC007684.2	0.462125	3.45E-23	postive
DBT	AC007684.2	0.438807	8.08E-21	postive
GCSH	AC010331.1	0.433838	2.45E-20	postive
DBT	AL390067.1	0.50342	7.41E-28	postive
ATP7A	AL031666.3	0.442046	3.88E-21	postive
DBT	AL031666.3	0.537425	3.29E-32	postive
ATP7A	AC079160.1	0.487334	5.81E-26	postive
DBT	AC079160.1	0.532814	1.37E-31	postive
ATP7A	Z99127.3	0.419989	4.90E-19	postive
DBT	Z99127.3	0.506684	2.97E-28	postive
ATP7A	AC078852.2	0.426935	1.11E-19	postive
DBT	AC078852.2	0.477253	7.96E-25	postive
LIAS	AL133410.1	0.406421	8.09E-18	postive
ATP7A	AC007365.1	0.485127	1.04E-25	postive
DBT	AC007365.1	0.470427	4.47E-24	postive

ATP7A	SDCBP2-AS1	0.457921	9.53E-23	postive
GCSH	SDCBP2-AS1	0.407544	6.44E-18	postive
ATP7A	AL121574.1	0.407764	6.16E-18	postive
DBT	AL121574.1	0.487947	4.94E-26	postive
NLRP3	SUGCT-AS1	0.424618	1.83E-19	postive
ATP7A	AP001469.2	0.445256	1.86E-21	postive
NLRP3	COL4A2-AS1	0.410152	3.79E-18	postive
ATP7A	C1orf220	0.434634	2.05E-20	postive
ATP7A	KLF7-IT1	0.464441	1.96E-23	postive
DBT	HID1-AS1	0.444838	2.05E-21	postive
ATP7A	DENND6A-AS:	0.417492	8.29E-19	postive
DBT	FARP1-AS1	0.461141	4.38E-23	postive
ATP7A	AC018766.1	0.46511	1.67E-23	postive
ATP7A	LINC02820	0.403606	1.42E-17	postive
DBT	LINC02820	0.420751	4.17E-19	postive
ATP7A	AC007314.1	0.441705	4.19E-21	postive
GCSH	AC063948.1	0.404631	1.16E-17	postive
ATP7A	KDM4A-AS1	0.47289	2.41E-24	postive
DBT	KDM4A-AS1	0.556606	6.85E-35	postive
ATP7A	MCM3AP-AS1	0.4402	5.90E-21	postive
ATP7A	AC008119.1	0.486254	7.72E-26	postive
ATP7A	AC131934.1	0.472096	2.94E-24	postive
DBT	AC002347.1	0.460438	5.20E-23	postive
GCSH	AC092123.1	0.493819	1.03E-26	postive
ATP7A	AC136604.2	0.419533	5.39E-19	postive
NLRP3	AC019254.1	0.417891	7.62E-19	postive
ATP7A	IQCH-AS1	0.441236	4.66E-21	postive
LIAS	IQCH-AS1	0.450441	5.60E-22	postive
DBT	IQCH-AS1	0.412616	2.28E-18	postive
DBT	AC022784.6	0.553527	1.90E-34	postive
DBT	AL021878.4	0.444209	2.37E-21	postive
ATP7A	AC103703.1	0.400068	2.88E-17	postive
DBT	AC073957.3	0.42364	2.25E-19	postive
DBT	AL732437.3	0.419197	5.79E-19	postive
ATP7A	AC078852.1	0.430367	5.26E-20	postive
DBT	AC078852.1	0.466189	1.28E-23	postive
ATP7A	AC040934.1	0.412334	2.42E-18	postive
LIAS	STX18-AS1	0.558196	4.03E-35	postive
ATP7A	AP001469.1	0.413643	1.85E-18	postive
ATP7A	OPA1-AS1	0.493631	1.08E-26	postive
DBT	OPA1-AS1	0.400686	2.55E-17	postive
DBT	AC010201.2	0.42306	2.55E-19	postive
ATP7B	LINC00456	0.538983	2.02E-32	postive
ATP7A	LINC00456	0.413024	2.10E-18	postive
DBT	LINC00456	0.562001	1.12E-35	postive
ATP7A	AC004918.3	0.522596	2.99E-30	postive
ATP7A	AC008750.4	0.472117	2.92E-24	postive
ATP7A	AC004832.5	0.433571	2.60E-20	postive
DBT	AC004832.5	0.418734	6.38E-19	postive
DBT	AC084781.1	0.450594	5.40E-22	postive
ATP7A	AC092828.1	0.42383	2.16E-19	postive
DBT	AC092828.1	0.532515	1.50E-31	postive
ATP7A	AC009716.1	0.422458	2.90E-19	postive
ATP7A	AC004837.4	0.508706	1.68E-28	postive
DBT	AC004837.4	0.587732	1.25E-39	postive
DBT	AC010789.2	0.511446	7.69E-29	postive
DBT	AC107027.3	0.439285	7.25E-21	postive
DBT	AP005262.1	0.424209	2.00E-19	postive

DBT	AC112493.1	0.495541	6.46E-27	postive
DBT	LINC02156	0.417246	8.73E-19	postive
ATP7A	AC010422.4	0.464742	1.82E-23	postive
DBT	AC104459.1	0.414648	1.50E-18	postive
ATP7A	AC106820.5	0.427715	9.37E-20	postive
DBT	AC106820.5	0.46972	5.33E-24	postive
ATP7A	Z94721.3	0.504946	4.84E-28	postive
DBT	Z94721.3	0.534137	9.12E-32	postive
ATP7A	AC138393.3	0.492528	1.46E-26	postive
LIAS	SNHG4	0.402886	1.64E-17	postive
DBT	AL139041.1	0.476586	9.44E-25	postive
ATP7A	Z98885.3	0.506085	3.51E-28	postive
DBT	Z98885.3	0.450473	5.56E-22	postive
ATP7A	AL391840.3	0.499798	2.02E-27	postive
ATP7A	LINC00339	0.42829	8.27E-20	postive
DBT	LINC00339	0.430317	5.31E-20	postive
DBT	AL592043.1	0.492262	1.56E-26	postive
ATP7A	SEPTIN7-DT	0.512954	4.99E-29	postive
DBT	AC006001.2	0.462782	2.94E-23	postive
ATP7A	AL139099.2	0.424897	1.72E-19	postive
ATP7A	AC012358.1	0.403777	1.38E-17	postive
LIAS	AC012358.1	0.40313	1.57E-17	postive
ATP7A	AL590133.1	0.403432	1.47E-17	postive
GCSH	AL590133.1	0.423069	2.55E-19	postive
DBT	CASC20	0.475777	1.16E-24	postive
ATP7A	AC104984.5	0.465371	1.56E-23	postive
ATP7A	AC073130.2	0.46027	5.41E-23	postive
DBT	AC073130.2	0.402669	1.72E-17	postive
ATP7A	AC022893.3	0.468999	6.37E-24	postive
DBT	AC022893.3	0.500504	1.66E-27	postive
ATP7A	TXNDC12-AS1	0.46123	4.29E-23	postive
DBT	TXNDC12-AS1	0.431654	3.96E-20	postive
ATP7A	GARS1-DT	0.475599	1.21E-24	postive
DBT	GARS1-DT	0.503511	7.22E-28	postive
ATP7A	AC025287.3	0.404466	1.20E-17	postive
GCSH	AC025287.3	0.423172	2.49E-19	postive
ATP7A	AC093535.1	0.418445	6.78E-19	postive
DBT	AC093535.1	0.545814	2.32E-33	postive
ATP7A	AC107419.1	0.420545	4.35E-19	postive
DBT	AC107419.1	0.581415	1.26E-38	postive
ATP7A	AC022558.3	0.411147	3.09E-18	postive
ATP7A	AC092171.1	0.469669	5.40E-24	postive
GCSH	AC127024.5	0.45877	7.77E-23	postive
GCSH	TMED2-DT	0.448915	7.99E-22	postive
ATP7A	AC004596.1	0.487573	5.45E-26	postive
ATP7A	AC037487.2	0.482207	2.22E-25	postive
ATP7A	GSTCD-AS1	0.476388	9.93E-25	postive
DBT	AC016571.1	0.414219	1.64E-18	postive
ATP7A	AC009090.6	0.533937	9.70E-32	postive
DBT	AC009090.6	0.470047	4.91E-24	postive
ATP7A	AC024075.3	0.451337	4.54E-22	postive
DBT	AC024075.3	0.435172	1.82E-20	postive
DBT	LINC00885	0.454185	2.32E-22	postive
ATP7A	AC005993.1	0.402368	1.82E-17	postive
ATP7A	AC073517.1	0.430157	5.50E-20	postive
DBT	AC073517.1	0.411036	3.16E-18	postive
ATP7A	NUTM2B-AS1	0.445567	1.73E-21	postive
DBT	NUTM2B-AS1	0.534915	7.18E-32	postive

ATP7A	AL139021.2	0.432062	3.62E-20	postive
DBT	AL139021.2	0.479801	4.14E-25	postive
ATP7A	AC234775.2	0.539858	1.54E-32	postive
ATP7A	AC139887.1	0.472255	2.83E-24	postive
ATP7A	AC106845.1	0.404604	1.17E-17	postive
DBT	AC106845.1	0.55783	4.56E-35	postive
ATP7A	AC211433.1	0.44718	1.20E-21	postive
DBT	AC211433.1	0.450819	5.12E-22	postive
ATP7A	AC004837.2	0.437625	1.05E-20	postive
DBT	AC004837.2	0.414452	1.56E-18	postive
